# *Jwa* participates the maintenance of intestinal epithelial homeostasis via ERK/FBXW7-mediated NOTCH1/PPARγ/STAT5 axis and acts as a novel putative aging related gene

**DOI:** 10.7150/ijbs.72751

**Published:** 2022-08-29

**Authors:** Xiong Li, Jingwen Liu, Yan Zhou, Luman Wang, Yifan Wen, Kun Ding, Lu Zou, Xia Liu, Aiping Li, Yun Wang, Heling Fu, Min Huang, Guoxian Ding, Jianwei Zhou

**Affiliations:** 1 Department of Molecular Cell Biology & Toxicology, Center for Global Health, School of Public Health, Nanjing Medical University, Nanjing 211166, China.; 2 Key Laboratory of Modern Toxicology of Ministry of Education, School of Public Health, Nanjing Medical University, Nanjing, 211166, China.; 3 Jiangsu Key Lab of Cancer Biomarkers, Prevention and Treatment, Collaborative Innovation Center for Cancer Medicine, Nanjing Medical University, Nanjing 211166, China.; 4 Animal Core Facility of Nanjing Medical University, Jiangsu Animal Experimental Center of Medical and Pharmaceutical Research, Nanjing 211166, China.; 5 Department of Geriatrics, Division of Geriatric Endocrinology, The First Affiliated Hospital of Nanjing Medical University, Nanjing 210029, China.

**Keywords:** JWA, Intestinal epithelial homeostasis, Intestinal stem cell, Notch signal, Ubiquitination, Aging

## Abstract

The intestinal epithelium is a rapid self-renewal and regenerated tissue of which the structural integrity is beneficial for maintaining health. The integrity of intestinal epithelium depends on the balance of cell proliferation, differentiation, migration, and the function of intestinal stem cells, which declines due to genetic defect or aging. *Jwa* participates in multiple cellular processes; it also responds to oxidative stress and repairs DNA damage. However, whether *Jwa* plays a role in maintaining the homeostasis of intestinal renewal and regeneration is not clear. In the present study, we firstly described that the deletion of *Jwa* disturbed the homeostasis of intestinal epithelial renewal and regeneration. *Jwa* deficiency promoted NOTCH1 degradation in the ERK/FBXW7-mediated ubiquitin-proteasome pathway, thus disturbing the PPARγ/STAT5 axis. These mechanisms might partially contribute to the reduction of intestinal stem cell function and alteration of intestinal epithelial cell lineage distribution, finally suppressing the renewal and regeneration of intestinal epithelium. Moreover, our results also revealed that *Jwa* was a novel putative aging related gene.

## Introduction

The intestinal epithelium is the tissue with the largest surface area in the digestive tract, giving it an important role in food digestion and nutrient absorption 1. In addition, it is also the first defensive line of the body's immune system, which participates in the formation of the intestinal mucosal barrier to prevent harmful microbiomes and toxins in the lumen from entering the blood circulation, and the processes such as immune response and regulation 2-4. Therefore, the structural integrity of the intestinal epithelium is beneficial for maintaining health. On the contrary, loss of intestinal epithelial integrity due to tissue degeneration or injury usually causes various of physical disorders and diseases, such as malnutrition, liver diseases, autoimmune diseases, neurological diseases and metabolic diseases 5.

The precise regulation of tissue renewal and regeneration is an effective mechanism to prevent and slow down tissue degeneration 6. In mammals, the intestinal epithelium renews rapidly and usually turns over within 3-5 days, due to the organized cell proliferation, differentiation, migration, and apoptosis, as well as the regenerative capability of intestinal stem cells (ISCs) 1. However, these characteristics make intestinal epithelium more susceptible to external environmental factors like ionizing radiation. The high dose radiation exposure can induce intestinal epithelial injury followed by gastrointestinal syndrome (GIS). The ISCs often respond immediately and regenerate quickly to repair the injured intestinal epithelium 7. However, certain factors that negatively affect the ISCs maintenance, such as genetic defect and aging, can inevitably inhibit renewal and prevent regeneration of intestinal epithelium after injury 7, 8.

*Jwa* gene, also named ADP-ribosylation-like factor 6 interacting protein 5 (*Arl6ip5*), is initially cloned from human bronchial epithelial (HBE) cells induced by all-trans retinoic acid (ATRA). We have previously demonstrated that JWA is a microtubule-associated protein participating in multiple cellular processes like cell proliferation, differentiation, migration and apoptosis 9, 10. Our further studies have indicate that *Jwa* is an active environment-responsive gene responding to oxidative stress and participating in DNA single-strand damage repair 11, 12, and exerting neuroprotective role via alleviating oxidative stress and inhibiting inflammation in the mouse model of 1-Methyl-4-phenyl-1,2,3,6-tetrahydropyridine (MPTP) or paraquat-induced Parkinson's disease 13, 14. Given the characteristics of intestinal epithelium and the known functions of *Jwa* gene, we hypothesized that *Jwa* might play a critical role in maintaining intestinal epithelial renewal, responding to the stimulation of environmental factor such as X-ray to intestinal epithelium and regulating its regeneration after injury.

In this study, we demonstrated that *Jwa* is essential for the renewal and regeneration of intestinal epithelium and we also proposed it as a novel putative aging-related gene. Our mechanism exploration further suggested that *Jwa* regulated intestinal epithelial homeostasis partially through the ERK/FBXW7-mediated NOTCH1/PPARγ/STAT5 axis.

## Materials and Methods

### Mouse

Mouse carrying *flox* alleles for the exon 2 of *Jwa* gene (*Jwa^fl/fl^* mouse), *Jwa* knockout (*Jwa*^-/-^) and littermate wild type (*Jwa*^+/+^) mice were described in our previous study 15. *Vil1*-cre mouse on a C57BL/6 background was obtained from Shanghai Model Organisms Center (Shanghai, China). Wild type C57BL/6 mice were purchased from SLAC Laboratory Animal Co., Ltd (Shanghai, China). Intestinal epithelial *Jwa* deletion (*Jwa*^IEC -/-^) and littermate wild type (*Jwa*^IEC +/+^) mice were generated by cross-mating *Vil1*-cre mouse with *Jwa^fl/lf^* mouse. All mice were maintained in the Animal Core Facility of Nanjing Medical University in a specific pathogen-free (SPF) condition. The animal operations were approved by the Institutional Animal Care and Use Committee of Nanjing Medical University.

### SA-β-gal staining

Freshly isolated liver tissue was embedded in the Tissue-Tek O.C.T compound (Sakura, Tokyo, Japan) and quick-frozen on dry ice. The 10 μm thickness frozen section was prepared on the CM1950 cryostat (Leica Biosystem, Wetzlar, Germany) and stained using a Senescent cell β-galactosidase staining kit (Servicebio, Wuhan, China) according to the manufacturer's guidelines.

### Oral glucose tolerance test

Mouse was orally administrated with 6 mg/kg body weight d-glucose (Macklin, Shanghai, China) dissolved in saline. The blood glucose levels at indicated time points (0, 15, 30, 60, 90 and 120 min) were measured using a handheld blood glucometer (Sinocare, Changsha, China) through tail tips.

### *In vitro* intestinal glucose absorption assay

*In vitro* intestinal glucose absorption assay was conducted according to previous study 16. In brief, mouse was fasted for 12 h and euthanized. The intestine was flushed with 37 °C pre-warmed Krebs-Ringer Bicarbonate HEPES (KRBH) buffer (Phygene, Fuzhou, China). A 2 cm segment of jejunum was isolated and injected with 300 μl 37 °C pre-warmed KRBH buffer contained 30 mM d-glucose, the two ends of the segment were tied with suture line. The KRBH buffer contained glucose in the segment was fully collected after incubated in cell incubator for 90 min. The segment was lysed with Cell and Tissue Lysis Buffer for Glucose Assay (Beyotime, Shanghai, China), another jejunum segment not injected with KRBH was used for the basal glucose assay. Glucose contents in the collected KRBH buffer and tissue lysate were measured using the Glucose Assay Kit (Beyotime) according to the manufacturer's guidelines. The consumed glucose in KRBH buffer was calculated and the absorption rated was obtained by dividing with total glucose in KRBH buffer.

### D-xylose absorption and excretion assay

D-xylose is a five-carbon monosaccharide that is absorbed by enterocytes only relied on the intact intestinal mucosa rather than digestive enzymes. It is neither metabolized *in vivo* nor re-absorbed by the renal tubules, and is excreted unchanged in the urine. Therefore, the D-xylose absorption and excretion test is a reliable method for evaluating intestinal absorption 17. The assay was based on previous reports with modifications 17-19. In brief, mouse was fasted for 4 h and orally administrated with 200 mg/kg body weight d-xylose (Macklin) dissolved in saline, the blood was collected from the post-ocular vein 1 h later, and serum was separated by centrifugating. Meanwhile, urine from mouse was continuous collected for 5 h. D-xylose concentrates in serum and urine were measurement using the D-xylose Assay Kit (Solarbio, Beijing, China) according to manufacturer's protocol. Total d-xylose in urine was calculated, and the excretion rate was obtained by dividing with total oral administrated d-xylose.

### BrdU^+^ cell migration assay

Mouse was intraperitoneally injected with 100 mg/kg bromodeoxyuridine (BrdU, APExBIO, Houston, TX, USA) dissolved in saline. The jejunum was isolated and fixed in 4% paraformaldehyde 72 h after injection. The BrdU^+^ cells were stained by immunofluorescence using the BrdU antibody.

### Intestinal villi and crypts isolation

Mouse intestine was isolated and flushed with phosphate-buffered saline (PBS) supplemented with penicillin/streptomycin. The jejunum was opened longitudinally and scraped by coverslip to separate the villus composition, which was washed several times in PBS with penicillin/streptomycin. The remained jejunum was washed several times in PBS and cut into 5 mm pellets, crypts were isolated by shaking the pellets violently in PBS supplemented with 5 mM ethylene diamine tetra acetic acid (EDTA) and filtered through 70 μm cell strainers (NEST, Wuxi, China).

### X-ray exposure

Mouse was anesthetized and placed into the RS 2000 Pro X-ray irradiator (Rad Source Technologies, Buford, GA, USA) to receive X-ray exposure (10 Gy, 1.5 Gy/min), the exposure area was limited to the whole abdomen by the control of built-in beam limiter in the instrument. Mouse was sacrificed on the indicated days after irradiation, the blood and intestine were collected for further experiment.

### Intestinal permeability assay

Mice were given 15 mg fluorescein isothiocyanate isomer-dextran 4 kDa (FD4, Sigma-Aldrich, St. Louis, MO, USA) by gavage. The serums were collected 4 h after FD4 administration. The serum fluorescence intensities (Excitation: 485 nm, Emission: 528 nm) were detected using the Infinite M200 PRO fluorescence microplate reader (TECAN, Männedorf, Switzerland). The concentration of FD4 was calculated via a standard curve prepared with serial dilutions of FD4.

### Histology, immunohistochemistry, and immunofluorescence

Isolated tissue was fixed in 4% paraformaldehyde and embedded in paraffin. The 6 μm thickness section was prepared on the Microm HM 340 E rotary microtome (Thermo Scientific, Waltham, MA, USA), and dewaxed in xylene and serial concentrations of ethanol before staining. For hematoxylin-eosin (H&E) and Alcian blue staining, the dewaxed section was stained using the H&E staining kit or Alcian blue staining kit (Servicebio) according to the manufacturer's protocol. For immunohistochemistry, the dewaxed section was boiled in Tris-EDTA antigen retrieval buffer (0.01 M Tris, 1 mM EDTA, 0.05% Tween 20, pH 9.0) for 10 min and incubated with the primary antibody (Supplementary Table S1) at 4 ºC overnight after being blocked with 5% bovine serum albumin (BSA). Then the section was incubated with horseradish peroxidase (HRP)-labeled secondary antibody and stained using the Diaminobenzidine (DAB) Color Development Kit (Servicebio). The section was permanently mounted using the glycerol jelly mounting medium (Servicebio) after dehydrated and clarified in ethanol and xylene. For immunofluorescence, the section was incubated with Alexa Fluor dye-labeled secondary antibody and mounted using the ProLong Diamond Antifade Mountant with DAPI (Thermo Scientific). The images were obtained using the Pannoramic MIDI digital slide scanner system (3DHISTECH, Budapest, Hungary).

### Proteomics analysis

Freshly isolated mouse jejunum was flushed with PBS, quick-frozen and stored in liquid nitrogen pending process, 3 independent jejunum samples were isolated for each genotype. Total protein was extracted by the lysis buffer (8 M urea and 1% protease inhibitors), protein concentration was determined using a bicinchoninic acid (BCA) assay kit (Beyotime). 100 μg protein per sample was labeled using a TMT10plex Isobaric Mass Tagging Kit (Thermo Scientific) according to the manufacturer's instructions. The labeled mixture was separated by reversed-phase high-performance liquid chromatography (RP-HPLC) using an Agilent 300extend C18 column (5 μm, 4.6 mm × 250 mm) to obtain peptide fractions. Peptides were separated by ultra HPLC, injected a NSI ion source for ionization, and analyzed on the Q-Exactive Plus mass spectrometry (Thermo Scientific). The raw data was processed by the Proteome Discoverer software (Thermo Scientific) based on the NCBI Protein Reference Sequence Database (txid10090, *Mus musculus*). Proteins with fold changes exceeding 1.5 or below 0.67 were defined as significantly up-regulation or down-regulation. The proteomics analysis was performed by CapitalBio Technology (Beijing, China).

### Cell culture, transfection, and treatment

Rat small intestinal epithelial crypt (IEC-6) cell (Zhong Qiao Xin Zhou, Shanghai, China) was cultured in Dulbecco's modified eagle medium (DMEM) supplemented with 10% fetal bovine serum (FBS, TransGen Biotech, Beijing, China), 100 U/ml penicillin, 0.1 mg/ml streptomycin (Beyotime) and 10 μg/ml recombinant human insulin (Zhong Qiao Xin Zhou). For cell transfection, small hairpin RNA (shRNA) plasmids for *Jwa* (Corues, Nanjing, China), small interfering RNA (siRNA) for *Fbxw7* (GenPharma, Shanghai, China) and their nonspecific control were synthesized (Supplementary Table S2), the commercial *Stat5a* overexpression (*Stat5a* OE) and *Hes1* overexpression (*Hes1* OE) plasmids were purchased (Youbio, Changsha, China). The plasmids and siRNA were transfected or co-transfected for 48 h using the Lipo8000 transfection reagent (Beyotime) following the manufacturer's protocol. To activate the phosphorylation of ERK1/2 or suppress the expression of PPARγ, the cell was treated with 10 μM Pamoic acid (PA, Macklin) or 10 μM GW9662 (MedChemExpress, Monmouth Junction, NJ, USA) for 24 h. For the Chx-chase assay, the cell was treated with 100 μg/ml cycloheximide (Chx, Selleck) at the indicated time points.

### EdU incorporation assay

5-ethynyl-2'-deoxyuridine (EdU) incorporation assay was conducted using an EdU Cell Proliferation Kit with Alexa Fluor 555 (Beyotime). In brief, a total of 10^5^ cells were seeded in 96 well cell culture plate after transfection and followed by compound treatment. Cells were incubated with 10 μM EuU for 2 h, then were fixed in 4% paraformaldehyde and permeabilized in 0.3% Triton X-100/PBS solution. EdU and nuclear staining was performed according to the manufacturer's instructions. The images were obtained on a Nikon Ti microscope (Nikon, Tokyo, Japan), EdU positive cell and nuclear were counted for the calculation of cell proliferation percentage.

### Immunoblotting

Total protein was extracted using the RIPA lysis buffer (50 mM Tris, 150 mM NaCl, 1% Triton X-100, 1% sodium deoxycholate, 0.1% SDS, pH 7.4) supplemented with 1% protease and phosphatase inhibitor cocktail (NCM, Suzhou, China) and quantified using the BCA kit. Protein (20-40 μg/lane) was separated by sodium dodecyl sulfate-polyacrylamide gel electrophoresis (SDS-PAGE) and blotted onto polyvinylidene fluoride (PVDF) membrane. The membrane was probed with diluted primary antibody (Supplementary Table S1) at 4 °C overnight after being blocked in 5% non-fat powder milk. The blot was detected using an Enhanced Chemiluminescence (ECL) detection kit (Vazyme, Nanjing, China) on the Chemiluminescence Image Analysis System (Tanon, Shanghai, China) after the HRP-labeled secondary antibody incubation.

### Quantitative reverse transcription and polymerase chain reaction (QRT-PCR)

Total mRNA was isolated using the RNAiso Plus reagent (Takara, Beijing, China) and reverse transcribed using the HiScript II 1st Strand cDNA Synthesis Kit with gDNA wiper (Vazyme). The PCR reaction procedure was carried out on the ABI 7900HT Real-Time PCR System (Applied Biosystems, Carlsbad, CA, USA) with the AceQ qPCR SYBR Green Master Mix (Vazyme) following the manufacturer's instruction. The synthetic cDNA was used as the template in the reaction, the primer pairs were listed in Supplementary Table S3.

### *In vitro* ubiquitination assay

The cell was transfected with ubiquitin plasmid and treated with 10 μM MG132 (Selleck) for 6 h. Total protein was then isolated using RIPA lysis buffer. Approximately 500 μg/250 μl protein per sample was incubated with the NOTCH1 antibody at 4 °C overnight and followed by incubated with Protein A/G Plus-Agarose (Beyotime) at 4 °C overnight. After being washed in PBS and centrifuged, the ubiquitin level in the precipitation was detected by immunoblotting.

### Statistical analysis

Data was analyzed on GraphPad Prism 8.0 software with the two-tail independent samples student's *t* test for two-group independent data, one-way analysis of variance (ANOVA) followed by Dunnett's *t* test for multiple-group independent data, log-rank test for survival curve analysis, and Fisher's exact test for the cases of survival and death. The number of sample (n) for animal experiments, and the number of independent replicates for cell experiments were indicated in the figure legends. Data was presented as mean with standard deviation (mean ± SD) in the figures. *P* < 0.05 was defined as the statistical difference.

## Results

### *Jwa* is a new putative aging-related gene in mice

**Figure 1:**
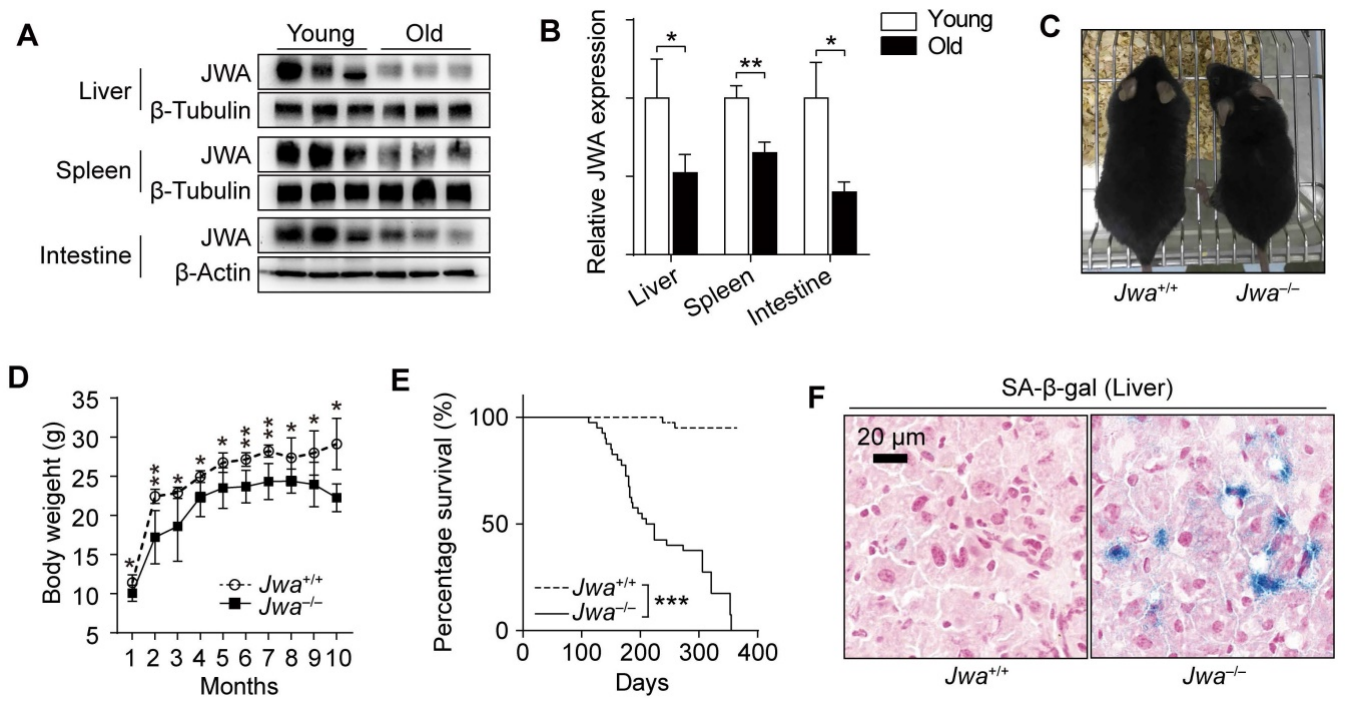
***Jwa* is a new putative aging-related gene in mice. (A, B)** Immunoblotting of JWA **(A)** and relatively JWA levels **(B)** in the liver, spleen and intestine of young (2-month-old, n=3) and old (24-month-old, n=3) mice.** (C)** Representative photograph of 6-month-old *Jwa*^+/+^ and *Jwa*^-/-^ mice. **(D)** Body weight curve of *Jwa*^+/+^ and *Jwa*^-/-^ mice from 1- to 10-month-old, n=6 for each genotype.** (E)** Survival curve of *Jwa*^+/+^ and *Jwa*^-/-^ mice, n=40 for each genotype. **(F)** SA-β-gal staining in the liver sections of 6-month-old *Jwa*^+/+^ and *Jwa*^-/-^ mice. *P < 0.05, **P < 0.01 and ***P < 0.001.

In the process of exploring *Jwa*, we firstly found that the old (24-month) mice had lower JWA levels in spleen, liver and intestine than young (2-month) mice (Fig. 1A, B). Subsequently, we observed that *Jwa*^-/-^ mice exhibited thinner bodies (Fig. 1C) at 6-month-old and lower body weight than littermate wild type (*Jwa*^+/+^) mice starting from 4-week-old (Fig. 1D). Moreover, the survival curves depicted that the *Jwa*^-/-^ mice had significantly lower survival rate than *Jwa*^+/+^ mice (Fig. 1E). Furthermore, there were visible senescence-associated β-galactosidase (SA-β-gal) positive cells in the liver section from 6-month-old *Jwa*^-/-^ mice rather than *Jwa*^+/+^ mice (Fig. 1F), indicating the cellular senescence. Our results preliminarily suggested that *Jwa* was a new putative aging-related gene.

### *Jwa* deletion disrupts intestinal epithelial homeostasis and impedes its development in early life

**Figure 2:**
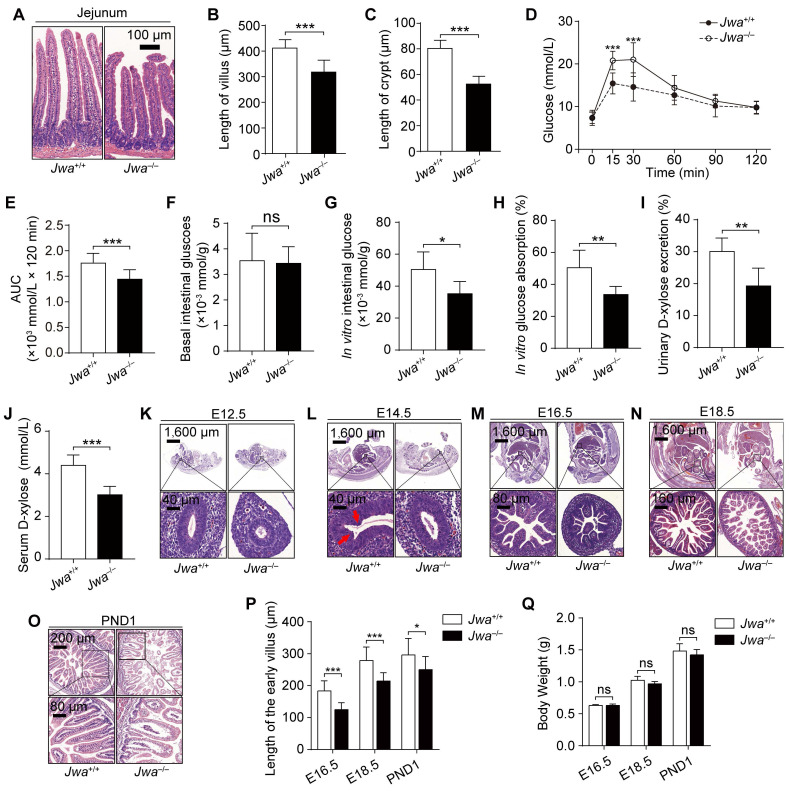
***Jwa* deletion disrupts intestinal epithelial homeostasis and impedes its development in early life. (A-C)** H&E staining of the jejunum sections **(A)**, the length measurement of villi **(B)** and crypts **(C)** in 6-month-old *Jwa*^+/+^ and *Jwa*^-/-^ mice, n=3 for each genotype.** (D, E)** OGTT curve **(D)** and the AUC measurement **(E)** in 6-month-old *Jwa*^+/+^ and *Jwa*^-/-^ mice, n=12 for each genotype. **(K-O)** H&E staining of the mice embryo at E12.5 **(K)**, E14.5 **(L)**, E16.5 **(M)**, E18.5 **(N)** and the newborn mice at PND1 **(O)**; the red arrows in E14.5 show the original folds of villus. **(P, Q)** Length of intestinal villi **(P)** and body weight **(Q)** of mice at E16.5, E18.5 and PND1, n=3 for each genotype. ^ns^ No significance, *P < 0.05, and ***P < 0.001.

We explored the histomorphology of the principal organs of mice and found that the primary alteration due to *Jwa* deletion was the atrophy of intestinal epithelium without any changes in other organs or tissues (Fig. 2A and Supplementary Fig. S1), which showed the reduced length of intestinal villi and crypts (Fig. 2B, C). To evaluate the intestine epithelium absorption, we performed the oral glucose tolerance test (OGTT) and found that *Jwa*^-/-^ mice had lower blood glucose level than *Jwa*^+/+^ mice after glucose gavage (Fig. 2D, E), which preliminary revealed the reduction in absorptive function of intestinal epithelium due to *Jwa* deletion. However, this result might be influenced by *in vivo* factors such as insulin. Therefore, we performed an* in vitro* intestinal glucose absorption assay, results showed that there was no difference in the basal intestinal glucose levels between *Jwa*^+/+^ and *Jwa*^-/-^ mice after fasting (Fig. 2F). After *in vitro* absorption process, the intestine isolated from *Jwa*^-/-^ mice exhibited significantly lower glucose level (Fig. 2G) and absorption rate (Fig. 2H) than *Jwa*^+/+^ mice. Moreover, results of d-xylose absorption and excretion test also showed that *Jwa*^-/-^ mice had lower serum d-xylose level (Fig. 2I) and urine d-xylose excretion rate (Fig. 2J) than *Jwa*^+/+^ mice. These results reminded us of the probable alterations of intestinal epithelial structure and function due to the JWA deletion. Furthermore, we observed that *Jwa* deletion delayed the development of intestinal epithelium in early life of mice, manifested by sluggish villi formation. In brief, on embryo day 12.5 (E12.5), the *Jwa*^-/-^ mice embryo showed thinner stratified epithelium than *Jwa*^+/+^ embryo (Fig 2K); on E14.5, the *Jwa*^+/+^ embryo showed early villus folds rather than the *Jwa*^-/-^ embryo (Fig 2L); on E16.5 (Fig. 2M), E18.5 (Fig. 2N) and postnatal day 1 (PND1) (Fig. 2O), the *Jwa*^-/-^ embryo and newborn mice showed shorter villi than *Jwa*^+/+^ embryo and newborn mice (Fig. 2P). However, the body weight on E16.5, E18.5 and PND1 showed no significantly difference between *Jwa*^+/+^ and *Jwa*^-/-^ embryo or newborn mice (Fig. 2Q). All above results suggested that *Jwa* played a critical role in intestinal epithelial homeostasis maintenance.

### JWA expresses higher in intestinal crypts than villi

**Figure 3:**
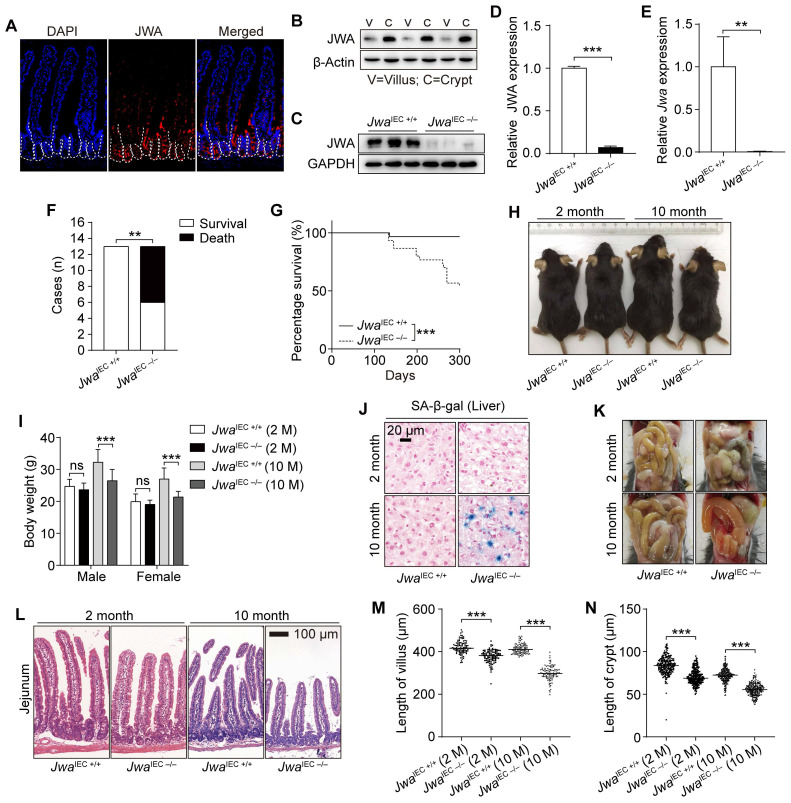
** Intestinal epithelial *Jwa* deletion affects intestinal epithelial homeostasis and promotes mice aging. (A)** Immunofluorescence staining of JWA in the intestine section of 2-month-old wild type mice. **(B)** Immunoblotting of JWA in intestinal villi and crypts of 2-month-old wild-type mice, n=3, “V” represents “villi” and “C” represents “crypts”.** (C, D)** Immunoblotting of JWA **(C)** and relatively JWA levels **(D)** in crypts of 2-month-old *Jwa*^IEC +/+^ and *Jwa*^IEC -/-^ mice, n=3 for each genotype.** (E)** QRT-PCR detection of *Jwa* levels in crypts of 2-month-old *Jwa*^IEC +/+^ and *Jwa*^IEC -/-^ mice, n=3 for each genotype.** (F)** Statistics of death and survival *Jwa*^IEC +/+^ and *Jwa*^IEC -/-^ mice by the end of 10-month-old, n=13 for each genotype at initial.** (G)** Survival curve of *Jwa*^IEC +/+^ and *Jwa*^IEC -/-^ mice by the end of 10-month-old, n=30 for each genotype.** (H)** Representative photograph of *Jwa*^IEC +/+^ and *Jwa*^IEC -/-^ mice at 2- and 10-month-old respectively.** (I)** Body weight of *Jwa*^IEC +/+^ and *Jwa*^IEC -/-^ mice at 2- and 10-month-old respectively, n=6-11 for each genotype.** (J)** SA-β-gal staining in the liver sections of *Jwa*^IEC +/+^ and *Jwa*^IEC -/-^ mice at 2- and 10-month-old. **(K)** Representative abdominal cavity images of *Jwa*^IEC +/+^ and *Jwa*^IEC -/-^ mice at 2- and 10-month-old.** (L-N)** H&E staining of the jejunum sections **(L)**, the length measurement of villi **(M)** and crypts **(N)** in *Jwa*^IEC +/+^ and *Jwa*^IEC -/-^ mice at 2-month-old and 10-month-old respectively, n=3 for each genotype. ^ns^ No significance, **P < 0.01 and ***P < 0.001.

Through The Human Protein Atlas database (https://www.proteinatlas.org), we got that the *Jwa* mRNA and JWA protein expressed widely in the human tissues and organs (Supplementary Fig. S2A, B). JWA protein was highest existing in the human duodenum and the whole small intestine (Supplementary Fig. S2B, C). We also detected that the JWA expresses in mouse intestine (Fig. 3A), especially the middle intestine (jejunum) (Supplementary Fig. S2D, E). Moreover, we observed that the JWA level was higher in intestinal crypts than villi (Fig. 3A, B). Therefore, we further hypothesized that *Jwa* might play a critical role in renewal and regeneration of intestinal epithelium, owing to the ISCs and niche located in the crypts, which are involved in the maintenance of intestinal epithelium.

### Intestinal epithelial *Jwa* deletion affects the homeostasis of the intestinal epithelium and promotes mice aging

To investigate the role of JWA on intestinal epithelial homeostasis maintenance, we constructed *Jwa*^IEC -/-^ and *Jwa*^IEC +/+^ mice (Supplementary Fig. S3A-C). We confirmed that *Jwa* was successfully deleted in crypt through RT-PCR, western blot and immunofluorescence (Fig. 3C-E and Supplementary Fig. S3D). In the mice cohort, we found that most of the *Jwa*^IEC -/-^ mice died before 10-month-old (Fig. 3F), and the 10-month survival rate of *Jwa*^IEC -/-^ mice was significantly lower than *Jwa*^IEC +/+^ mice (Fig. 3G). Like *Jwa^-/-^* mice, *Jwa*^IEC -/-^ mice also showed thinner bodies, lighter weights and more SA-β-gal positive senescent cells in liver than *Jwa*^IEC +/+^ mice. However, these phenotypes only manifested at 10-month-old, rather than 2-month-old (Fig. 3H-J). We dissected the euthanized mice and found that the 10-month-old *Jwa*^IEC -/-^ mice also showed intestinal congestion and bloat signs (Fig. 3K). We then observed that *Jwa*^IEC -/-^ mice had shorted villi and crypts than *Jwa*^IEC +/+^ mice, especially at 10-month-old (Fig. 3L-N). Our results suggested that intestinal epithelial *Jwa* deletion could disrupt intestinal epithelial homeostasis. Interestingly, intestinal epithelial *Jwa* deletion also appeared to partially contribute to mouse aging.

### Intestinal epithelial *Jwa* deletion inhibits cell proliferation and reduces intestinal stem cells in crypt

**Figure 4:**
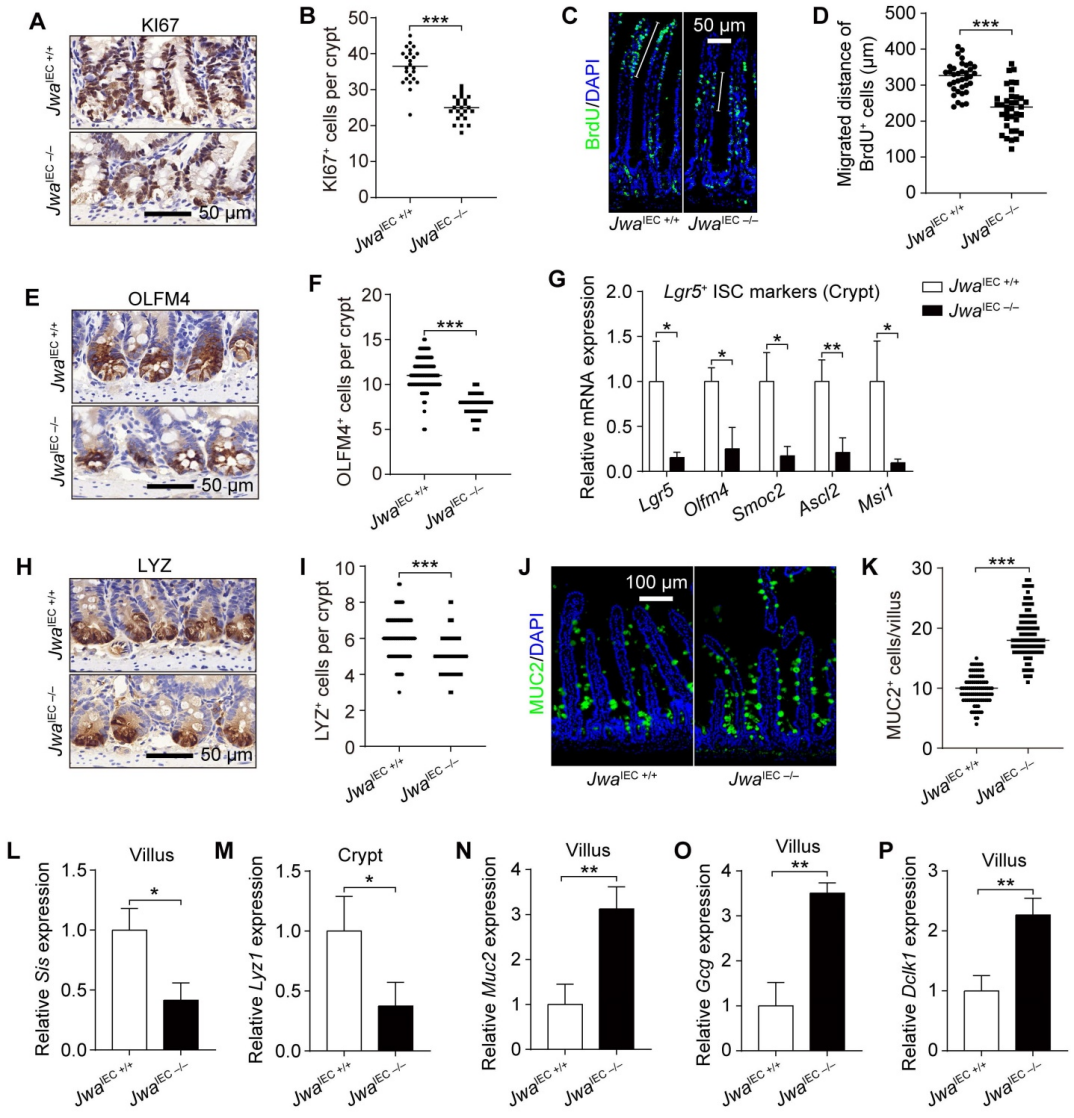
** Intestinal epithelial *Jwa* deletion reduces intestinal stem cells and skews the distributions of intestinal epithelial cells lineage. (A, B)** Immunochemistry staining of KI67 in the intestinal sections **(A)** and KI67 positive cell counts in crypts **(B)** of 2-month-old *Jwa*^IEC +/+^ and *Jwa*^IEC -/-^ mice, n=3 for each genotype. **(C, D)** Immunofluorescence staining of BrdU in the intestinal sections **(C)** and BrdU positive cells migration measurement **(D)** of 2-month-old *Jwa*^IEC +/+^ and *Jwa*^IEC -/-^ mice, n=3 for each genotype. **(E, F)** Immunochemistry staining of OLFM4 in the intestinal sections **(E)** and OLFM4 positive cell counts in crypts **(F)** of 2-month-old *Jwa*^IEC +/+^ and *Jwa*^IEC -/-^ mice, n=3 for each genotype. **(G)** QRT-PCR detection of ISC markers in crypts of 2-month-old *Jwa*^IEC +/+^ and *Jwa*^IEC -/-^ mice, n=3 for each genotype. **(H, I)** Immunochemistry staining of LYZ in the intestinal sections **(H)** and LYZ positive cell counts in crypts **(I)** of 2-month-old *Jwa*^IEC +/+^ and *Jwa*^IEC -/-^ mice, n=3 for each genotype. **(J, K)** Immunofluorescence staining of MUC2 in the intestinal sections **(J)** and MUC2 positive cell counts in villI **(K)** of 2-month-old *Jwa*^IEC +/+^ and *Jwa*^IEC -/-^ mice, n=3 for each genotype. **(L-P)** QRT-PCR detection of absorption enterocytes marker *Sis* **(L)** in villi, Paneth cell marker *Lyz1* **(M)** in crypts, goblet cell marker *Muc2* **(N)**, enteroendocrine cell marker *Gcg* **(O)**, and tuft cell marker *Dclk1* in villi **(P)** of 2-month-old *Jwa*^IEC +/+^ and *Jwa*^IEC -/-^ mice, n=3 for each genotype. *P < 0.05, **P < 0.01 and ***P < 0.001.

Intestinal epithelial integrity depends on the proliferation, differentiation, migration of intestinal epithelial cells (IECs) and function of ISCs. The KI67 staining showed intestinal epithelial *Jwa* deletion inhibited the proliferation of crypt cells (Fig. 4A, B and Supplementary Fig. S4A). It also hindered the intestinal epithelium renewal, revealed by BrdU^+^ cell migration assay (Fig. 4C, D). We next found that intestinal epithelial *Jwa* deletion markedly reduced the ISCs number at the intestinal crypts, revealed by the decrease of OLFM4 positive cells (Fig. 4E, F and Supplementary Fig. S4B) and reduction of OLFM4 expression (Supplementary Fig. S4C, D). Moreover, reduced mRNA levels of ISCs markers such as *Lgr5*, *Olfm4*, *Smoc2*, *Ascl2*, and *Msi1* were detected in crypts from *Jwa*^IEC -/-^ mice than *Jwa*^IEC +/+^ mice (Fig. 4G). These results further suggested that intestinal epithelial* Jwa* deletion might suppress the renewal and regeneration of intestinal epithelium through inhibiting cell proliferation and reducing ISCs in crypt.

### Intestinal epithelial *Jwa* deletion skews the distributions of intestinal epithelial cell lineages

The distribution balance of IEC lineages also plays an essential role in intestinal epithelial homeostasis maintenance. To explore the impact of intestinal epithelial* Jwa* deletion on IEC lineages, we examined the levels of various IEC markers. Results showed markedly decreased LYZ positive Paneth cells number in crypts (Fig. 4H, I and Supplementary Fig. S4G), elevated MUC2 and Alcian blue positive Goblet cells in villi (Fig. 4J, K and Supplementary Fig. S4E, F) due to intestinal epithelial *Jwa* deletion. Moreover, the mRNA levels of several mature IEC markers further revealed the disordered composition of intestinal cell types caused by intestinal epithelial *Jwa* deletion. Such as lower absorptive enterocyte markers (Fig. 4L and Supplementary Fig. S4H) in villi and Paneth cell markers (Fig. 4M and Supplementary Fig. S4I) in crypts; higher goblet cell markers (Fig. 4N and Supplementary Fig. S4J), enteroendocrine cell markers (Fig. 4O and Supplementary Fig. S4K), and tuft cell markers (Fig. 4P and Supplementary Fig. S4L) in villi. Our results suggested intestinal epithelial *Jwa* deletion might lead to differentiation of secretory cell lineage, but inhibited the differentiation of Paneth cells and absorptive cell lineage.

### Intestinal epithelial *Jwa* deletion exacerbates the injury and prevents intestinal epithelial regeneration after X-ray irradiation exposure

**Figure 5:**
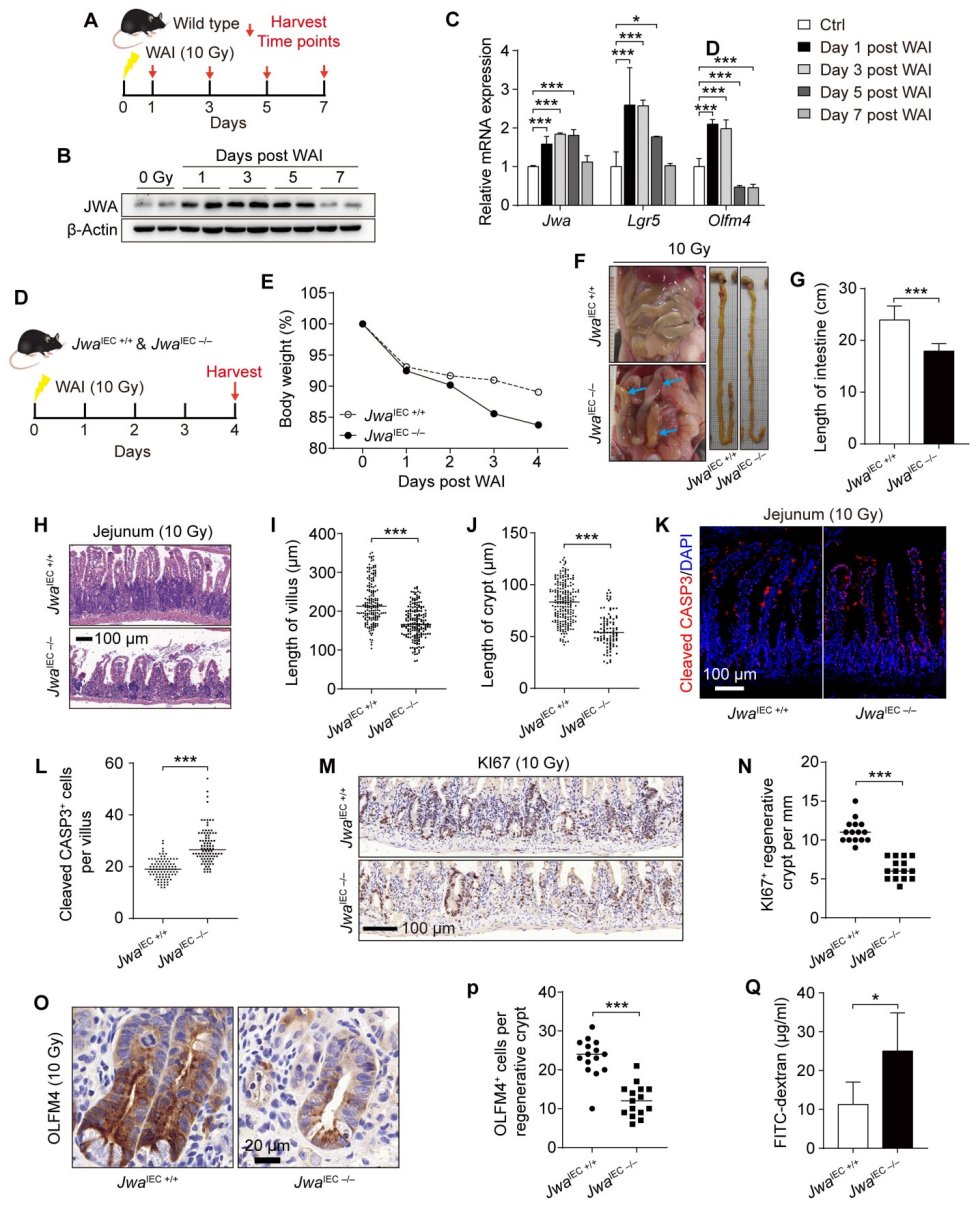
** Intestinal epithelial *Jwa* deletion exacerbates injury and prevents regeneration of intestinal epithelium after X-ray exposure. (A)** 2-month-old wild type mice are exposed to WAI and sacrificed at indicated time points. **(B)** Immunoblotting of JWA in crypts of the wild-type mice exposure to WAI. **(C)** QRT-PCR detection of *Jwa* and the ISCs marker *Lgr5* and *Olfm4* levels in crypts of the wild-type mice exposure to WAI, n=6 for each time point. **(D)** 2-month-old *Jwa*^IEC +/+^ and *Jwa*^IEC -/-^ mice are exposed to WAI and sacrificed at the indicated time point. **(E)** Bodyweight curve of *Jwa*^IEC +/+^ and *Jwa*^IEC -/-^ mice exposure to WAI, n=6 for *Jwa*^IEC +/+^ and n=5 for *Jwa*^IEC -/-^ mice. **(F)** Representative abdominal cavity and intestinal images of *Jwa*^IEC +/+^ and *Jwa*^IEC -/-^ mice exposure to WAI, blue arrows show the intestinal hyperemia and swelling. **(G)** Intestinal length of *Jwa*^IEC +/+^ and *Jwa*^IEC -/-^ mice exposure to WAI, n=6 for each genotype. **(H-J)** H&E staining of the jejunum sections **(H)**, the length measurement of villi **(I)** and crypts **(J)** of *Jwa*^IEC +/+^ and *Jwa*^IEC -/-^ mice exposure to WAI, n=3 for each genotype. **(K, L)** Immunofluorescence staining of Cleaved CASP-3 in the intestinal sections **(K)** and Cleaved CASP-3 positive cell counts in villi **(L)** of *Jwa*^IEC +/+^ and *Jwa*^IEC -/-^ mice exposure to WAI. **(M, N)** Immunochemistry staining of KI67 in the intestinal sections **(M)** and KI67 positive crypts counts in the epithelium **(N)** of *Jwa*^IEC +/+^ and *Jwa*^IEC -/-^ mice exposure to WAI, n=3 for each genotype. **(O, P)** Immunochemistry staining of OLFM4 in the intestinal sections **(O)** and OLFM4 positive cells counts in the regenerative crypts **(P)** of *Jwa*^IEC +/+^ and *Jwa*^IEC -/-^ mice exposure to WAI, n=3 for each genotype. **(Q)** Permeability of intestinal epithelium to FD4 in *Jwa*^IEC +/+^ and *Jwa*^IEC -/-^ mice exposure to WAI, n=6 for each genotype. *P < 0.05 and ***P < 0.001.

To investigate the regeneration capability of intestinal epithelium, we subjected the wild type mice to whole abdomen X-ray irradiation (WAI) at a dose of 10 Gy. We sacrificed the mice at the indicated time points after WAI and collected crypts for later analysis (Fig. 5A). We saw the rising of *Jwa* in both mRNA and protein levels during the recovery period within five days after WAI, and returned to base levels on the seventh day (Fig. 5B, C). In addition, interestingly, the mRNA levels of ISCs markers *Olfm4* and *Lgr5* altered consistently with *Jwa* (Fig. 5C). These further prompted us that *Jwa* participated in the ISCs regeneration driven intestinal epithelial repairment. We then exposed both *Jwa*^IEC +/+^ and *Jwa*^IEC -/-^ mice to WAI, and analyzed at the fourth day (Fig. 5D). As we expected, intestinal epithelial JWA deletion made mice more susceptible to WAI, exacerbated the injury and prevented the regeneration of intestinal epithelium. The body weights of *Jwa*^IEC -/-^ mice lost faster than *Jwa*^IEC +/+^ mice within the four days after WAI (Fig. 5E). We dissected the mice and found that *Jwa*^IEC -/-^ mice showed visible intestinal hyperemia and swelling and markedly shorter intestine rather than *Jwa*^IEC +/+^ mice (Fig. 5F, G). We also observed worse damaged crypts and villi (Fig. 5H-J) and more cleaved CASP3 positive apoptotic cells (Fig. 5K, L) in intestine sections from *Jwa*^IEC -/-^ mice, these results revealed more severe intestinal epithelial injury in *Jwa*^IEC -/-^ mice than *Jwa*^IEC +/+^ mice. Moreover, we found fewer KI67 positive regenerative crypts (Fig. 5M, N) and fewer OLFM4 positive cells (Fig. 5O, P) in regenerative crypts owing to intestinal epithelial *Jwa* deletion. Finally, we performed the intestinal epithelial permeability assay in mice after WAI and found that the intestinal epithelial of *Jwa*^IEC -/-^ mice were more permeable to FD4 than *Jwa*^IEC +/+^ mice (Fig. 5Q). These results suggested intestinal epithelial *Jwa* deletion exacerbated the injury and prevented intestinal epithelial regeneration after WAI.

### *Jwa* deficiency disturbs PPARγ/STAT5 axis through inhibiting Notch signal pathway

**Figure 6:**
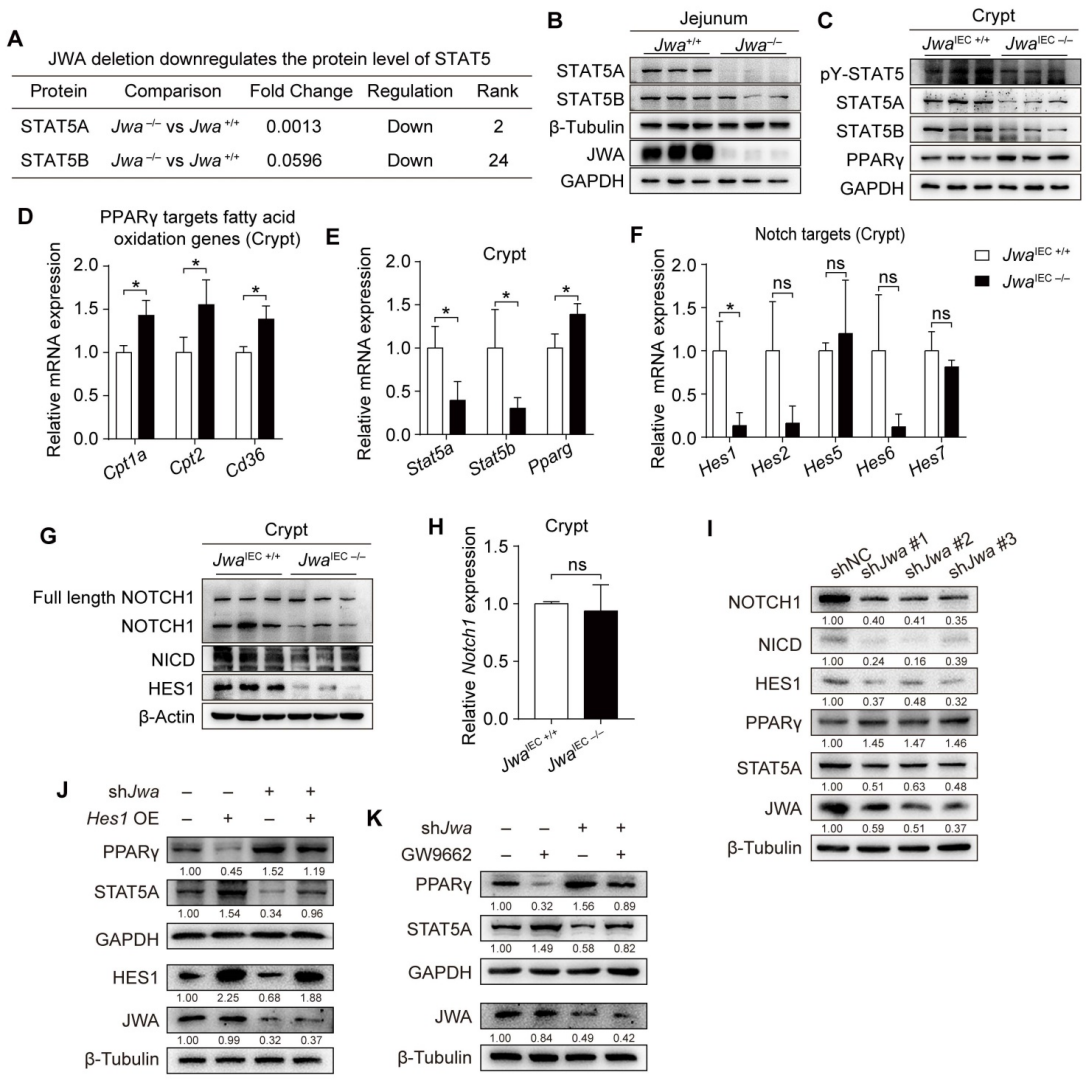
** Intestinal epithelial *Jwa* deletion disturbs PPARγ/STAT5 axis through inhibiting Notch signal pathway. (A)** Screen of the down-regulated proteins Stat5 from the proteomics analysis performed on the jejunum of 2-month-old *Jwa*^+/+^ and *Jwa*^-/-^ mice, n=3 for each genotype. **(B)** Immunoblotting of STAT5A, STAT5B and JWA in the jejunum of 2-month-old *Jwa*^+/+^ and *Jwa*^-/-^ mice. **(C)** Immunoblotting of PPARγ, STAT5A, STAT5B, and pY-STAT5 in crypts of 2-month-old *Jwa*^IEC +/+^ and *Jwa*^IEC -/-^ mice. **(D)** QRT-PCR detection of the PPARγ targets fatty acid oxidation genes *Cpt1a*, *Cpt2* and *Cd36* in crypts of 2-month-old *Jwa*^IEC +/+^ and *Jwa*^IEC -/-^ mice, n=3 for each genotype. **(E)** QRT-PCR detection of *Stat5a*, *Stat5b* and *Pparg* levels in crypts of 2-month-old *Jwa*^IEC +/+^ and *Jwa*^IEC -/-^ mice, n=3 for each genotype. **(F)** QRT-PCR detection of Notch target genes *Hes1*, *Hes2*, *Hes5*, *Hes6* and *Hes7* in crypts of 2-month-old *Jwa*^IEC +/+^ and *Jwa*^IEC -/-^ mice, n=3 for each genotype. **(G)** Immunoblotting of full-length NOTCH1, NOTCH1, NICD and HES1 in crypts of 2-month-old *Jwa*^IEC +/+^ and *Jwa*^IEC -/-^ mice. **(H)** QRT-PCR detection of *Notch1* levels in crypts of 2-month-old *Jwa*^IEC +/+^ and *Jwa*^IEC -/-^ mice, n=3 for each genotype. **(I)** Immunoblotting of JWA, STAT5A, PPARγ, HES1, NICD AND NOTCH1 in IEC-6 cells transfected with sh*Jwa* plasmids, 3 independent replicates were carried out. **(J)** Immunoblotting of JWA, STAT5A, PPARγ AND HES1 in IEC-6 cells co-transfected with sh*Jwa* and *Hes1* OE plasmids, 3 independent replicates were carried out. **(K)** Immunoblotting of JWA, STAT5A AND PPARγ in IEC-6 cells transfected with *shJwa* plasmid followed by GW9662 (10 μM) treatment, 3 independent replicates were carried out. ^ns^ No significance and *P < 0.05.

To explore *Jwa* deletion-induced molecule alterations, we distinguished the differential proteins in jejunum tissues between *Jwa*^+/+^ and *Jwa*^-/-^ mice through proteomics analysis (Supplementary Table S4). We discovered that *Jwa* deletion distinctively down-regulated the protein levels of signal transducer and activator of transcription 5 (STAT5) (Fig. 6A), which was intimately related to the maintenance of ISCs and intestinal epithelium. We then determined the reduced levels of STAT5 in both jejunum from *Jwa*^-/-^ mice (Fig. 6B and Supplementary Fig. S5A, B) and crypts from *Jwa*^IEC -/-^ mice (Fig. 6C and Supplementary S5C, D). However, phosphorylation ratio of STAT5 in crypts showed no difference between *Jwa*^IEC +/+^ and *Jwa*^IEC -/-^ mice (Fig. 6C and Supplementary S5E), so did the phosphorylation ratio of JAK2 (Supplementary Fig. S5F, G), the upstream kinase of STAT5. These results reminded that *Jwa* deficiency only affected the expression of STAT5 rather than phosphorylation. The nuclear transcription factor peroxisome proliferator-activated receptor-gamma (PPARγ) has been reported to play a negative role on ISC maintenance, it also acts as a transcriptional repressor to supervise the expression of STAT5. Our results showed that *Jwa* deletion did elevate the level of PPARγ (Fig. 6C and Supplementary Fig. S5H) and the levels of PPARγ targeted fatty acid oxidation genes (Fig. 6D) in crypts, subsequently reduced the transcription levels of *Stat5a* and *Stat5b* (Fig. 6E). Interestingly, *Jwa* deletion also increased the transcription level of *Pparg* (Fig. 6E). We then searched the JASPAR database (https://jaspar.genereg.net/) for the potential transcript factors (TFs) binding to the promoter region of *Pparg*. We found that the primary TFs were the hairy and enhancer of splits (*Hes*), a target genes family of Notch signal (Supplementary Table S5). We quantified several *Hes* genes and found that *Jwa* deletion drastically reduced the mRNA levels of *Hes1* (Fig. 6F), a transcription repressor of *Pparg*; these results informed us that *Jwa* deletion might cause Notch signal inhibition. We then confirmed that *Jwa* deletion down-regulated the levels of NOTCH1 (i.e., the cleaved NOTCH1) (Fig. 6G and Supplementary Fig. S5I), NOTCH1 intracellular domain (NICD) (Fig. 6G and Supplementary Fig. S5J), and HES1 (Fig. 6G and Supplementary Fig. S5K) in crypts. However, the newly synthesized full-length NOTCH1 (Fig. 6G and Supplementary Fig. S5L) and the mRNA level of *Notch1* (Fig. 6H) showed no alterations. Furthermore, we knocked down *Jwa* in IEC-6 cells by transfecting with sh*Jwa* plasmid, and observed the similar molecule changes as *Jwa* deletion crypts (Fig. 6I), these changes in IEC-6 cells were reversed by *Hes1* over-expression (Fig. 6J) and the PPARγ antagonist GW9662 treatment (Fig. 6K). Our results suggested that *Jwa* deficiency disturbed PPARγ/STAT5 axis by inhibiting the Notch signal pathway.

### *Jwa* deficiency promotes ubiquitination degradation of Notch1 via ERK/FBXW7 axis

**Figure 7:**
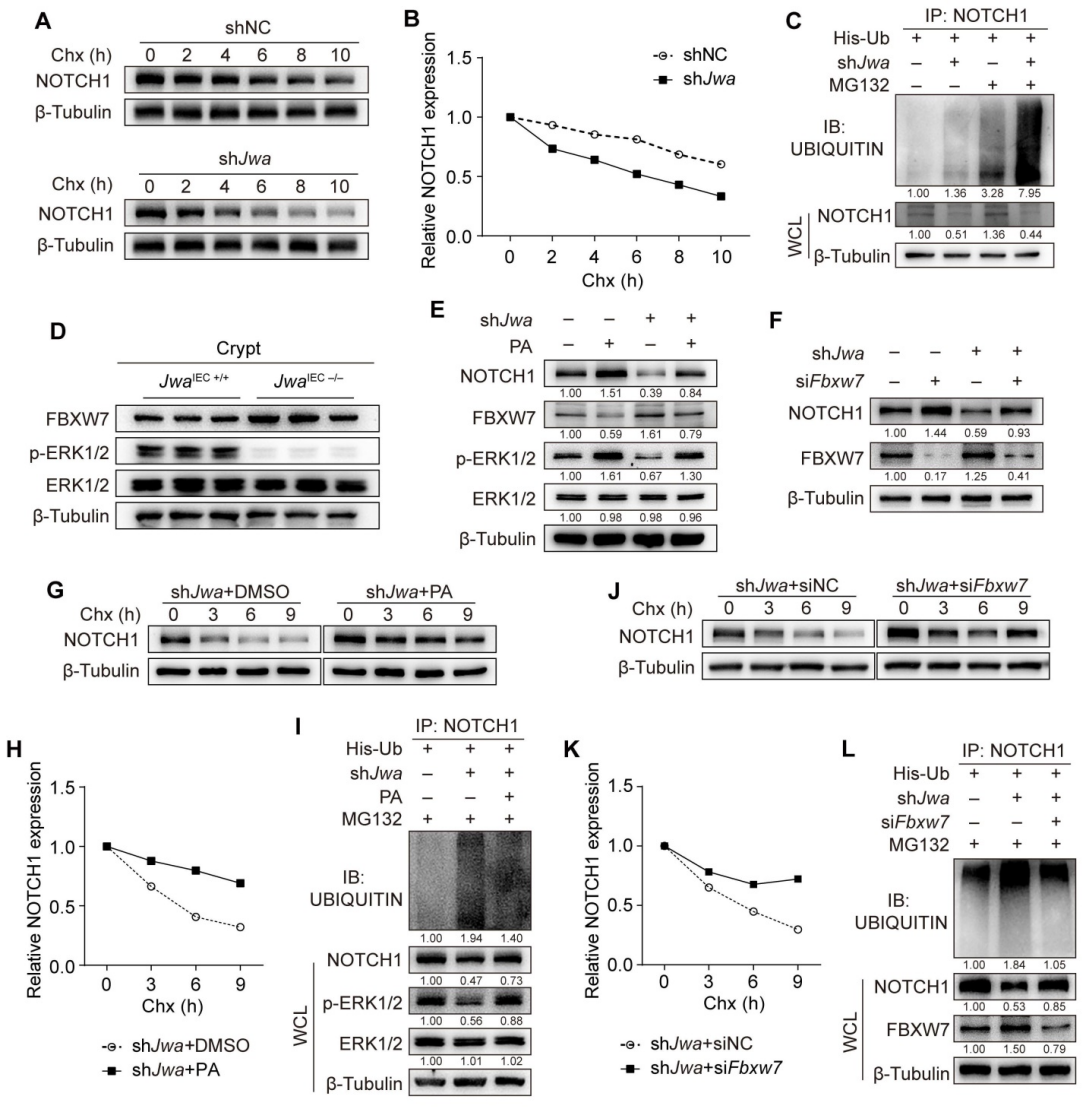
***Jwa* deficiency promotes ubiquitination degradation of NOTCH1 through ERK/FBXW7 axis. (A, B)** Chx-chase assay of NOTCH1 in IEC-6 cells transfected with sh*Jwa* plasmid, 3 independent replicates were carried out.** (C)** *In vitro* ubiquitination assay of NOTCH1 in IEC-6 cells transfected with sh*Jwa* plasmid, 3 independent replicates were carried out.** (D)** Immunoblotting of NOTCH1, FBXW7, ERK1/2 and p-ERK1/2 in crypts of 2-month-old *Jwa*^IEC +/+^ and *Jwa*^IEC -/-^ mice.** (E)** Immunoblotting of NOTCH1, FBXW7, ERK1/2 and p-ERK1/2 in IEC-6 cells transfected with sh*Jwa* plasmids followed by Pamoic Acid (10 μM) treatment, 3 independent replicates were carried out.** (F)** Immunoblotting of NOTCH1 and FBXW7 in IEC-6 cells co-transfected with sh*Jwa* plasmid and si*Fbxw7*, 3 independent replicates were carried out.** (G, H)** Chx-chase assay of NOTCH1 in IEC-6 cells transfected with sh*Jwa* plasmid followed by Pamoic Acid (10 μM) treatment, 3 independent replicates were carried out. **(I)** *In vitro* ubiquitination assay of NOTCH1 in IEC-6 cells co-transfected with sh*Jwa* followed by Pamoic Acid (10 μM) treatment, 3 independent replicates were carried out. **(J, K)** Chx-chase assay of NOTCH1 in IEC-6 cells co-transfected with sh*Jwa* plasmid and si*Fbxw7*, 3 independent replicates were carried out.** (L)** *In vitro* ubiquitination assays of NOTCH1 in IEC-6 cells co-transfected with sh*Jwa* and si*Fbxw7*, 3 independent replicates were carried out.

To verify the mechanism underlying the reduction of NOTCH1 caused by *Jwa* deficiency, we conducted the Chx-chase assay in IEC-6 cells. Results showed that *Jwa* deficiency accelerated NOTCH1 degradation (Fig. 7A, B). We then performed the *in vitro* ubiquitination assay and found that JWA deficiency promoted the degradation of NOTCH1 via ubiquitin-proteasome pathway (Fig. 7C). To gain insight into the reason for *Jwa* deficiency-induced NOTCH1 degradation, we predicted the potential E3 ubiquitin ligases acting on NOTCH1 through the UbiBrowser Database. We screened the top five high-confidence scored ligases (Supplementary Fig. S6A) and verified them in *Jwa* deficiency crypts and IEC-6 cells. However, *Jwa* deficiency did not increase their levels in crypts (Supplementary Fig. S6B-G) or cells (Supplementary Fig. S6H). We have previously confirmed *Jwa* as an upstream activator of the ERK1/2. Reportedly, ERK1/2 negatively regulates F-box and WD repeat domain containing-7 (FBXW7), a known E3 ubiquitin ligase targets multiple substrates including NOTCH1. FBXW7 is also a candidate in the predicted list although with low-confidence score. Therefore, we suspected that *Jwa* deficiency might promote degradation of NOTCH1 through ERK/FBXW7 axis. It was worth noting that we did observe that *Jwa* deficiency inhibited phosphorylation of ERK1/2 and increased the levels of FBXW7 in both crypts (Fig. 7D and Supplementary Fig. S6I, J) and cells (Supplementary Fig. S6K). To further clarify the role of the ERK/FBXW7 signal axis on *Jwa* deficiency-induced NOTCH1 degradation, we treated the cells with PA to activate the phosphorylation of ERK1/2. The results showed that ERK1/2 activation could effectively inhibit FBXW7 expression, reverse *Jwa* deficiency-induced NOTCH1 down-regulation and downstream molecular changes (Fig. 7E and Supplementary Fig. S6L). Meanwhile, we transfected the cells with si*Fbxw*7 and achieved the same consequent as PA treatment (Fig. 7F and Supplementary Fig. S6M). Furthermore, we performed the Chx-chase assay and the *in vitro* ubiquitination assay in PA treated or si*Fbxw*7 transfected cells. Results showed that PA treatment or si*Fbxw7* transfection could effectively enhance the stability of NOTCH1 and reduce the ubiquitination degradation of NOTCH1 caused by *Jwa* deficiency (Fig. 7G-L). Our results suggested that *Jwa* deficiency inhibited the phosphorylation of ERK1/2 and promoted NOTCH1 degradation through FBXW7-mediated ubiquitin-proteasome pathway.

### Restoration of the ERK/FBXW7 and NOTCH1/PPARγ/STAT5 axes reverses *Jwa* deficiency-induced cellular phenotypic changes *in vitro*

**Figure 8:**
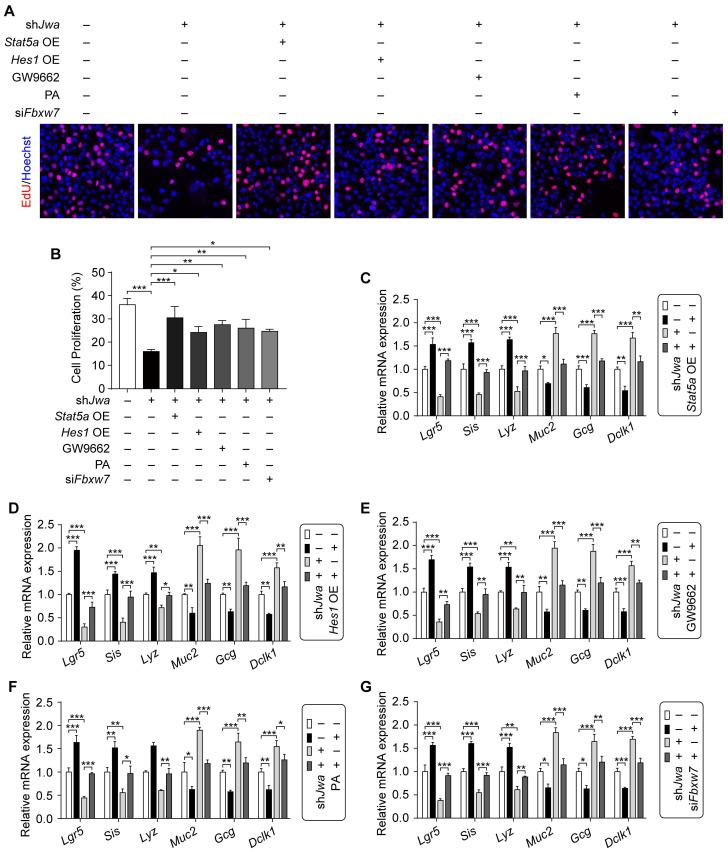
** Restoration of the ERK/FBXW7 and NOTCH1/PPARγ/STAT5 axes reverses *Jwa* deficiency-induced cellular phenotypic changes *in vitro*. (A, B)** EdU staining in IEC-6 cell transfected with sh*Jwa* plasmid, co-tranfected with sh*Jwa* and *Stat5a* OE plasmids, co-tranfected with sh*Jwa* and *Hes1* OE plasmids, transfected with sh*Jwa* plasmid followed by GW9662 (10 μM) treatment, transfected with sh*Jwa* plasmid followed by Pamoic acid (10 μM) treatment or co-tranfected with sh*Jwa* and si*Fbxw7* **(A)**, and the cell proliferation assay **(B)**, 3 independent replicates were carried out. **(C-G)** QRT-PCR detection of ISC marker *Lgr5*, absorptive enterocyte marker *Sis*, Paneth cell marker *Lyz1*, goblet cell marker *Muc2*, enteroendocrine cell marker *Gcg* and tuft cell marker *Dclk1* in IEC-6 cell co-tranfected with sh*Jwa* and *Stat5a* OE plasmids **(C)**, co-tranfected with sh*Jwa* and *Hes1* OE plasmids **(D)**, transfected with sh*Jwa* plasmid followed by GW9662 (10 μM) treatment **(E)**, transfected with sh*Jwa* plasmid followed by Pamoic acid (10 μM) treatment **(F)** or co-tranfected with sh*Jwa* and si*Fbxw7* **(G)**, 3 independent replicates were carried out. *P < 0.05, **P < 0.01 and ***P < 0.001.

Given the role of cell proliferation, intestinal stem cell and epithelial cell distribution in intestinal epithelial renewal and regeneration, we further conduced the in vitro assays to validate the effects of ERK/FBXW7 and NOTCH1/PPARγ/STAT5 axes on the changes of these issues caused by *Jwa* deficiency in IEC-6 cells. Results showed that *Jwa* deficiency significantly inhibited cell proliferation revealed by EdU incorporation assay in IEC-6 cells, which was rescued by *Stat5a*, *Hes1* overexpression, GW9662, PA treatment or si*Fbxw7* transfection (Fig. 8A, B and Supplementary Fig. S7A-E). Moreover, *Jwa* deficiency drastically reduced ISCs marker levels and altered the expression of mature IECs markers in IEC-6 cells, *Stat5a*, *Hes1* overexpression, GW9662, PA treatment or si*Fbxw7* transfection also reversed these phenotypes (Fig. 8C-G and Supplementary Fig. S7F-J). These results further revealed that JWA regulated cells proliferation, the expressions of ISC and mature IEC markers through ERK/FBXW7 and NOTCH1/PPARγ/STAT5 axes *in vitro*, these might indirectly expound that *Jwa* partially regulated cell proliferation, ISC maintenance and IEC distribution through this potential mechanism.

## Discussion

Intestinal epithelium is a rapid renewal tissue composed of villi and crypts. The proliferative ISCs that reside in the bottom of crypts are responsible for epithelial maintenance. ISCs generate the transit-amplifying progenitor cells that differentiate into mature IECs and migrate up to the villi to replace senescent or damaged IECs and complete epithelial renewal 20. These characteristics make the intestinal epithelium more susceptible to environmental factors such as ionizing radiation 7. Since the reduced ISCs activity, the intestinal epithelium regenerates more slowly in old mice than young mice after irradiation-induced injury 8, 21. The present study showed intestinal epithelial *Jwa* deletion drastically inhibited the proliferating of crypt cells and reduced the number of *Lgr5*^+^ ISCs, thus causing epithelial atrophy, which was more noticeable in elderly mice. Moreover, *Jwa* also responded to X-ray irradiation and participated in epithelial regeneration. Intestinal epithelial *Jwa* deletion aggravated injury and prevented regeneration of the epithelium in mice after 10 Gy WAI, suggesting that *Jwa* was a regulator for intestinal epithelial maintenance.

Multiple signaling transduction pathways, including Wnt, Notch, EGF, BMI, YAP/TAZ, etc., independently or cooperatively maintain intestinal epithelial homeostasis through ISC-dependent renewal and regeneration 22. JAK/STAT signaling pathway is a central node that regulates cellular processes through signal transduction 23. It responds to tissue turnover and regeneration via regulating stem cells, including hematopoietic stem cells 24, embryonic stem cells 25, as well as ISCs 26. It is also a maintainer for cancer stem cells and promoter for multiple cancers, such as colorectal cancer 27. STAT5, consisting of the transcription activator STAT5A and STAT5B, is a positive contributor to ISCs and intestinal epithelial regeneration in the dextran sulfate sodium (DSS)-induced colitis, *Clostridium difficile* infection-induced Ileocolitis, and irradiation-induced intestinal injury mouse models 28, 29. The present study showed that *Jwa* deficiency down-regulated STAT5 at both the transcription and translation levels, suggesting that *Jwa* maintained intestinal epithelium through STAT5 signaling.

PPARs, consist of three subtypes (α, δ/β, and γ), are nuclear hormone receptors activated by fatty acids and endogenous ligands, and responsible for lipid and glucose metabolism homeostasis 30, 31. PPARα and PPARδ/β is able to augment ISCs function in high-fat diet, aging and injury, and promotes intestinal tumorigenesis by activating fatty acid oxidation (FAO) 31, 32. PPARγ can regulate the proliferation and differentiation of intestinal progenitor cells through the region-specific promotion of FAO, and maintains intestinal epithelial renewal 20. Therefore, it is no doubt that FAO plays a supportive role in ISC maintenance 20, 31-33. However, on the other hand, high PPARγ activity also shows the inhibitory effect on Wnt/β-catenin signal pathway and impairs *Lgr5*^+^ ISCs function, although the FAO-related genes are induced 34. The present study showed that *Jwa* deficiency elevated both transcription and translation levels of PPARγ, and thus causing the elevating of FAO-related genes in crypt. Nonetheless, there were opposite effects of FAO-related genes induction on intestinal phenotypes when *Jwa* was deleted. This result suggested that *Jwa* might modulate intestinal phenotypes through other roles of PPARγ, such as its transcriptional regulatory functions. It is reported that PPARγ activation can inhibit various signals, including JAK/STAT 35. For example, activation of PPARγ can inhibit leukemia stem cells by suppressing STAT5 expression 36. In the present study, STAT5s were the pronounced down-regulated molecules due to *Jwa* deletion, therefore, we considered that the elevated PPARγ level due to *Jwa* deletion mainly exhibited a negative effect on intestinal phenotypes by inhibiting STAT5, whereas the PPARγ-induced FAO has a weak supportive effect. However, more sufficient evidences are required to explain why *Jwa* deletion-induced FAO has the opposite effect on intestinal phenotypes as previous studies.

The Notch signal pathway regulates cell proliferation, differentiation, tumorigenesis, and stem cell maintenance 27, 37. Transcription repressor *Hes1* is a target gene of Notch signal and plays an essential role in stem and progenitor cell maintenance; it is reported to prevent hematopoietic stem cell exhaustion by suppressing PPARγ expression 38, 39. Moreover, NOTCH/HES1 axis inhibition accompanied by PPARγ activation can repress the progression of colorectal cancer via suppressing cancer stem cells 40. Here we reported that *Jwa* deficiency inhibited Notch signal through ubiquitin-proteasome pathway-mediated degradation of the NOTCH1 receptor. The down-regulation of Notch target gene *Hes1* was also the reason for PPARγ activation due to *Jwa* deficiency. The E3-ubiquitin ligase FBXW7 targets multiple substrates such as C-JUN, C-MYC, and NOTCH1; it works as a tumor suppressor for colorectal cancer by inhibiting Notch signal. However, FBXW7 deletion alters the maintenance of intestinal stem/progenitor cells and the fate of differentiated cells and induces colorectal tumorigenesis via losing suppression on Notch signal in mice intestine 41, 42. The tumor suppressor FBXW7 expression is inversely correlated with ERK activation in pancreatic cancer, sustained activation of the RAS-RAF-MEK-ERK pathway phosphorylates FBXW7 at Thr205, and destabilizes FBXW7 43, 44. We previously demonstrated that JWA was an upstream activator of the ERK 45, 46. The present study showed that *Jwa* deficiency up-regulated FBXW7 expression by inhibiting the phosphorylation of ERK1/2, thus promoting NOTCH1 degradation via ubiquitin-proteasome pathway.

ERK1/2 is a bona fide factor in the regulation of stemness, proliferation and differentiation of stem cells, many studies also demonstrated that ERK1/2 positively regulated the proliferation of IECs. For example, dietary Glu accelerates intestinal epithelial renewal and gut growth through EGFR/ERK-promoted ISC proliferation in porcine intestine 47. Intestinal claudin-1 overexpression up-regulates the Notch signal via activating ERK1/2 signal, and promotes cell proliferation in both small intestine and colon epitheliums 48. The EGF family ligand NRG1 was shown to have a remarkable ability to increase the stemness and proliferation of ISCs by activating ERK1/2 phosphorylation 49. However, ERK1/2 deletion was shown to increase the KI67^+^ intestinal crypt cells, it appeared to be caused by ERK5 activation due to ERK1/2 deletion. When ERK5 was inhibited, the expression levels of both *Mki67* and* Lgr5* were both significantly reduced in ERK1/2-deficiency organoids; although they did not show any changes in the condition without ERK5 alteration, targeting both ERK1/2 and ERK5 showed inhibition of colorectal tumor cell proliferation, which was more pronounced when simultaneous suppression of ERK1/2 and ERK5 50. This suggests that ERK1/2 may exhibit bidirectional effects on the maintenance of intestinal epithelial homeostasis when regulated by different upstream signaling molecules or targeting different downstream molecules. JWA is also named as the Putative MAPK-activating protein PM27, perhaps including ERK5, which increase the proliferation of IECs. Taken together, we speculated that the *Jwa* ko-associated phenotypes are partially due to the contribution of the signaling axis downstream of ERK1/2, i.e., Notch signal and PPARγ/STAT5 axis. However, more evidence for the effects of JWA-mediated ERK signal on intestinal epithelial homeostasis should be further investigated.

Notch signal promotes the IECs differentiation into enterocyte lineage while antagonizing secretory cell fate in intestine 42, 51, which is reversed by FBXW7 deletion 42. The present study found that intestinal *Jwa* deletion promoted differentiated IECs into secretory cell lineages and suppressed the absorbing cell lineages. However, Paneth cells were reduced, presumably due to the suppression of Paneth cells by JWA deficiency-caused STAT5 down-regulation 29. Therefore, the typical phenotype of Notch signal inhibition, co-localization of Paneth cell marker LYZ and Goblet cell marker MUC2 in crypts 20, was not observed in *Jwa*-deficiency mice. The reason for *Jwa* deficiency-caused differential regulation on Paneth cells and other secretory cell lineages is an interesting issue worthy of further exploration.

Aging is one of the main factors of tissue degeneration, including the intestinal epithelium. Taking together our findings of long-term studies on the function of *Jwa*, such as oxidative stress response, DNA repair and neuroprotection 11-14., etc., hinting that *Jwa* may be associated with aging, which might also be revealed by decreased JWA levels in several organs due to aging and by shortened lifespan and elevated liver senescent cells due to *Jwa* deletion in the present study. Interestingly, the same phenotypes were also observed in intestinal epithelial *Jwa* deletion mice. Given that systemic and intestinal epithelial *Jwa* deletion both induced atrophy of intestinal epithelium and mice aging. Moreover, studies showed that the alterations in intestinal epithelial homeostasis could also influence the lifespan of lower creatures such as C. elegans and *Drosophila* 52, 53, making the intestine considered a potential target organ for anti-aging 54. Therefore, we hypothesized that intestinal epithelial atrophy might be one of the reasons for accelerated aging in mice, considering the long-term nutrient malabsorption through depauperated intestinal epithelium, although dietary restriction without malnutrition is beneficial for improving aging and aging-related diseases 55. However, the present study only preliminary elucidated of *Jwa* as a putative aging-related gene. How *Jwa* affects aging and whether the effect is related to the regulation of intestinal epithelial homeostasis requires further in-depth investigation. Moreover, in this study, the investigated mechanisms were partially contributed to the maintenance of intestinal epithelial homeostasis related to *Jwa*, however, there is no enough evidence whether targeting these mechanisms especially in intestine will altered the aging process.

**Figure 9:**
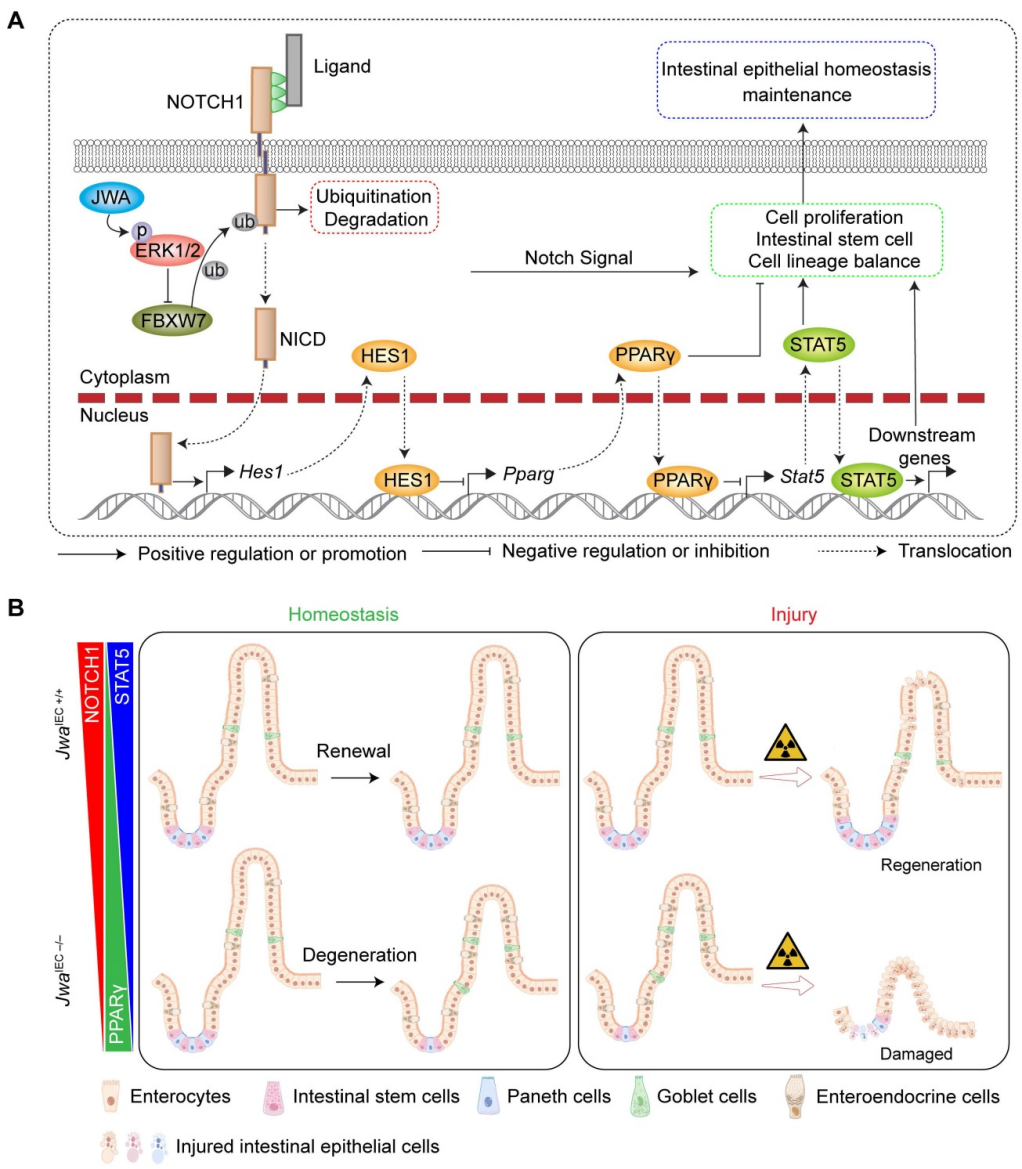
** Schematic overview for this study. (A)** The mechanism underlying *Jwa* regulating homeostasis of intestinal epithelium. **(B)** Intestinal epithelial JWA deletion disrupts homeostasis of intestinal epithelium in mice.

In summary, the present study shows that *Jwa* deficiency promoted NOTCH1 degradation via the ubiquitin-proteasome pathway in the ERK/FBXW7 dependent manner, thus disturbing the PPARγ/STAT5 axis, finally interfered with intestinal epithelial homeostasis by inhibiting cell proliferation, reducing intestinal stem cells, and altering epithelial cell distribution (Fig. 9A). However, the effects and restoring experiments of these mechanisms were only performed *in vitro* in the IEC-6 cell line, and more adequate *in vivo* experiments were required to demonstrate these proposed mechanisms. In one word, these mechanisms, as a consequence of *Jwa* deficiency, partially resulted in a restricted homeostasis of intestinal epithelial renewal and regeneration after injury (Fig. 9B). Furthermore, this study also preliminary proposed *Jwa* as a novel putative aging-related gene.

## Supplementary Material

Supplementary methods, figures and tables.

## Abbreviations

ISC: Intestinal stem cell
GIS: Gastrointestinal syndrome
BrdU: Bromodeoxyuridine
FD4: Fluorescein isothiocyanate isomer-dextran 4 kDa
shRNA: small hairpin RNA
siRNA: small interfering RNA
PA: Pamoic acid
Chx: Cycloheximide
SA-β-gal: Senescence-associated β-galactosidase
OGTT: Oral glucose tolerance test
*Olfm4*: Olfactomedin 4
*Lgr5*: Leucine-rich repeat containing G protein-coupled receptor 5
*Smoc2*: SPARC-related modular calcium-binding protein 2
*Ascl2*: Achaete-scute family BHLH transcription factor 2
*Msi1*: Musashi RNA binding protein 1
WAI: Whole abdomen X-ray irradiation
CASP3: CASPASE-3
ERK: Extracellular regulated protein kinases
STAT5: Signal transducer and activator of transcription 5
JAK2: Janus kinase 2
PPARγ: Peroxisome proliferator-activated receptor-gamma
*Hes1*: Hairy and enhancer of splits 1
NICD: NOTCH1 intracellular domain
FBXW7: F-box and WD repeat domain containing-7
LYZ: Lysozyme
MUC2: Mucin 2
*Sis*: sucrase isomaltase
*Lct*: Lactase
*Dpp4*: Dipeptidyl Peptidase 4
*Mmp7*: Matrix Metallopeptidase 7
*Spink4*: Serine Peptidase Inhibitor Kazal Type 4
*Tff3*: Trefoil Factor 3
*Gcg*: Glucagon
*Ngn3*: Neurogenin 3
*Reg4*: Regenerating Family Member 4
*Dclk1*: Doublecortin Like Kinase 1
*Hck*: HCK Proto-Oncogene, Src Family Tyrosine Kinase
