# Supplementary material for: Jwa participates the maintenance of intestinal epithelial homeostasis via ERK/FBXW7-mediated NOTCH1/PPARγ/STAT5 axis and acts as a novel putative aging related gene
Source: Int J Biol Sci. 2022 Aug 29;18(14):5503–21. doi: 10.7150/ijbs.72751 (PMC9461671; doi:10.7150/ijbs.72751)
Supplement: Supplementary file 1 — Supplementary methods, figures and tables. [file ijbsv18p5503s1.pdf]

Supplementary Materials

Jwa participates the maintenance of intestinal epithelial homeostasis via ERK/FBXW7-mediated NOTCH1/PPARγ/STAT5 axis and acts as a novel putative aging related gene

Xiong Li[1,2,3], Jingwen Liu[1,2,3], Yan Zhou[1,2,3], Luman Wang[1,2,3], Yifan Wen[1,2,3], Kun Ding[1,2,3], Lu Zou[1,2,3], Xia Liu[1,2,3], Aiping Li[1,2,3], Yun Wang[4], Heling Fu[4], Min Huang[5], Guoxian Ding[5] and Jianwei Zhou[1,2,3,*]

[1]Department of Molecular Cell Biology & Toxicology, Center for Global Health, School of Public Health, Nanjing Medical University, Nanjing 211166, China.

[2]Key Laboratory of Modern Toxicology of Ministry of Education, School of Public Health, Nanjing Medical University, Nanjing, 211166, China.

[3]Jiangsu Key Lab of Cancer Biomarkers, Prevention and Treatment, Collaborative Innovation Center for Cancer Medicine, Nanjing Medical University, Nanjing 211166, China.

[4]Animal Core Facility of Nanjing Medical University, Jiangsu Animal Experimental Center of Medical and Pharmaceutical Research, Nanjing 211166, China.

[5]Department of Geriatrics, Division of Geriatric Endocrinology, The First Affiliated Hospital of Nanjing Medical University, Nanjing 210029, China.

* Corresponding Author. E-mail: jwzhou@njmu.edu.cn (Jianwei Zhou)

Supplementary Method

Genotyping of intestinal epithelial *Jwa* deletion mice

Genomic DNA was isolated from mouse tail and the PCR procedure was performed using the Quick Genotyping Assay Kit for Mouse Tail (Beyotime). The primers used for genotyping were as followed: *flox* (Forward: 5'-CCACTGTTTCCTCTGTTG-3'; Reverse: 5'-GTGAAAACCACTGAGAACC-3'); *Vil1*-cre (Common Forward: 5'-GCCTTCTCCTCTAGGCTCGT-3', Wild type Reverse：5'-TATAGGGCAGAGCTGGAGGA-3', Mutant Reverse：5'-AGGCAAATTTTGGTGTACGG-3'), the products were separated by agarose gel electrophoresis and the images were obtained on the Gel Doc XR Gel Imaging System (Bio-Rad, Hercules, CA, USA). When genotyping for *flox*, image showed two bands in 341 and 437 bp indicating $Jwa^{fl/+}$ (Supplementary Fig. S3B, lane 1), one band in 437 bp indicating $Jwa^{fl/fl}$ (Supplementary Fig. S3B, lane 2), one band in 341 bp indicating $Jwa^{+/+}$ (Supplementary Fig. S3B, lane 3). When genotyping for *Vil1*-cre*,* image showed one band in 182 bp indicating wild type, two bands in 150 and 180 bp indicating *Vil1*-cre. The genotypes of mice were identified combining with the results of genotyping for *flox* and *Vil1*-cre. When the results of genotyping for *flox* and *Vil1*-cre were $Jwa^{fl/fl}$ and *Vil1*-cre respectively, the genotype of mouse was $Jwa^{fl/fl}$ *Vil1*-cre, i.e., the intestinal epithelial *Jwa* deletion mouse, we abbreviated it as $\mathrm{Jwa}^{IEC\ +/+}$. When the results of genotyping for *flox* and *Vil1*-cre were $Jwa^{fl/fl}$ and wild type respectively, the genotype of mouse was $Jwa^{fl/fl}$, i.e., the littermate wild type mouse, we abbreviated it as $\mathrm{Jwa}^{IEC\ -/-}$ (Supplementary Fig. S3A).

Supplementary Figures

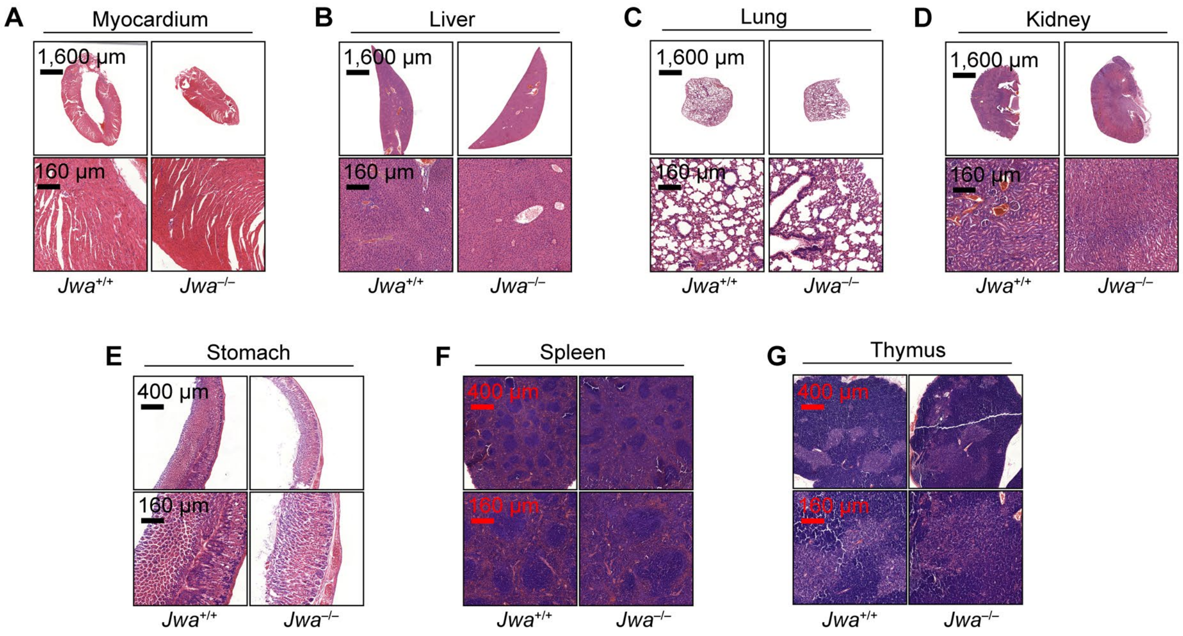

Supplementary Figure S1. No significant morphological difference is observed in the main organs of *Jwa*$^{+/+}$ and *Jwa*$^{-/-}$ mice. (A-G) H&E staining of the main organs or tissues such as myocardium **(A)**, liver **(B)**, lung **(C)**, kidney **(D)**, stomach **(E)**, spleen **(F)**, and thymus **(G)** of *Jwa*$^{+/+}$ and *Jwa*$^{-/-}$ mice at 16-month-old.

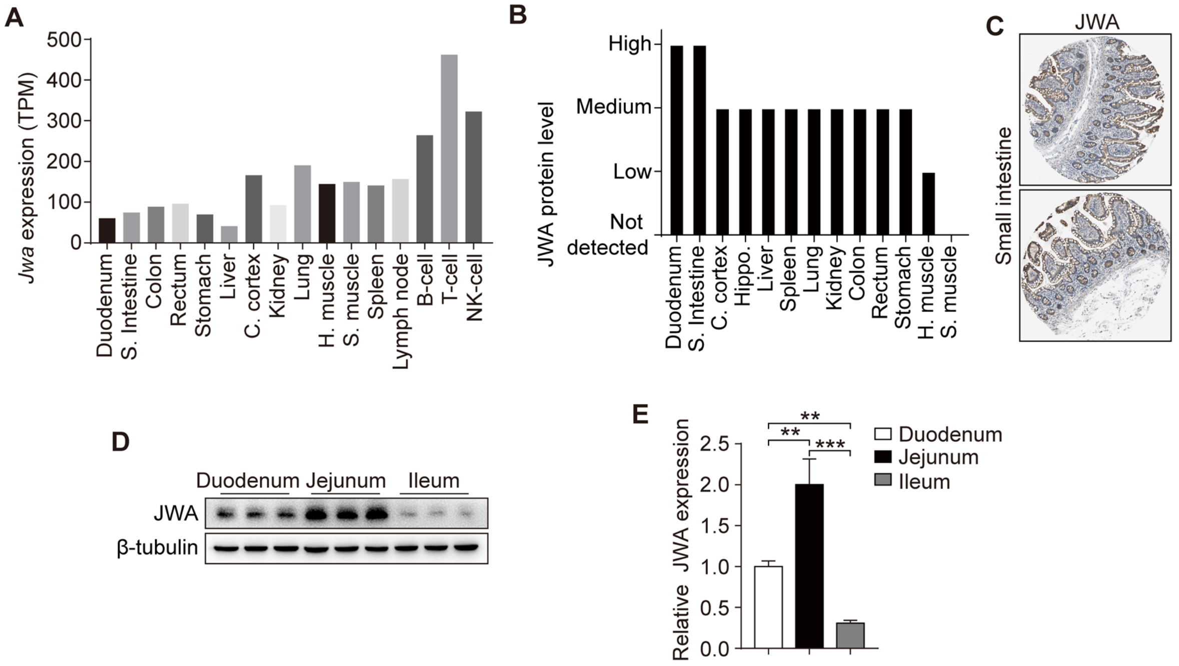

Supplementary Figure S2. The expression profile of JWA in tissues and organs. (A) *Jwa* mRNA levels in multiple tissues or organs, data was from the human protein atlas database (THPA database: https://www.proteinatlas.org); **(B)** JWA protein levels in multiple tissues or organs, data was from THPA database. **(C)** Immunochemistry images of JWA in the intestinal sections from THPA database. **(D, E)** Immunoblotting of JWA **(D)** and relatively JWA expression **(E)** in the duodenum, jejunum and ileum crypts of wild-type mice. ***P* < 0.01 and ****P* < 0.001.

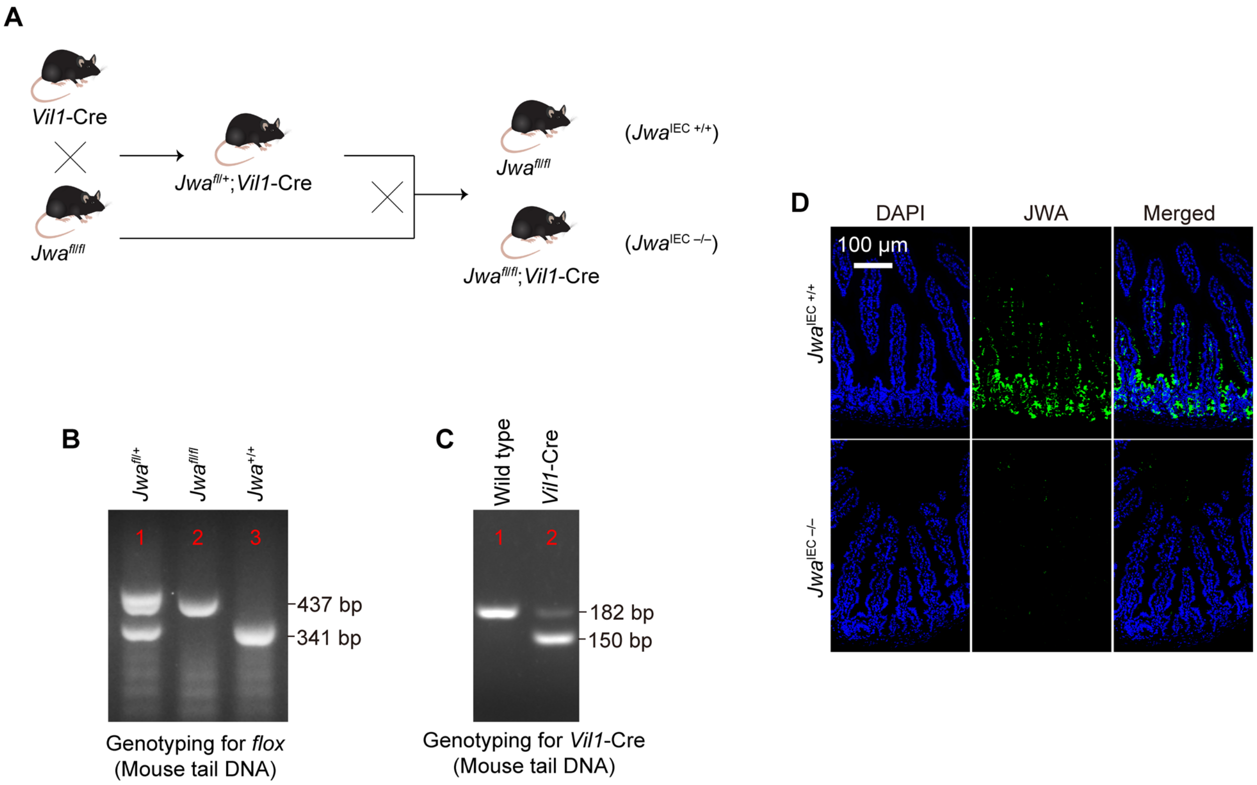

Supplementary Figure S3. Construction and genotyping of intestinal epithelial *Jwa* deletion mice. (A) Schematic diagram of mouse reproduction. **(B)** Image of genotyping for *flox* alleles. **(C)** Image of genotyping for *Vil1*-cre alleles. **(D)** Immunofluorescence of JWA in intestinal sections of 2-month-old *Jwa*$^{\mathrm{IEC}\ +/+}$ and *Jwa*$^{\mathrm{IEC}\ -/-}$ mice.

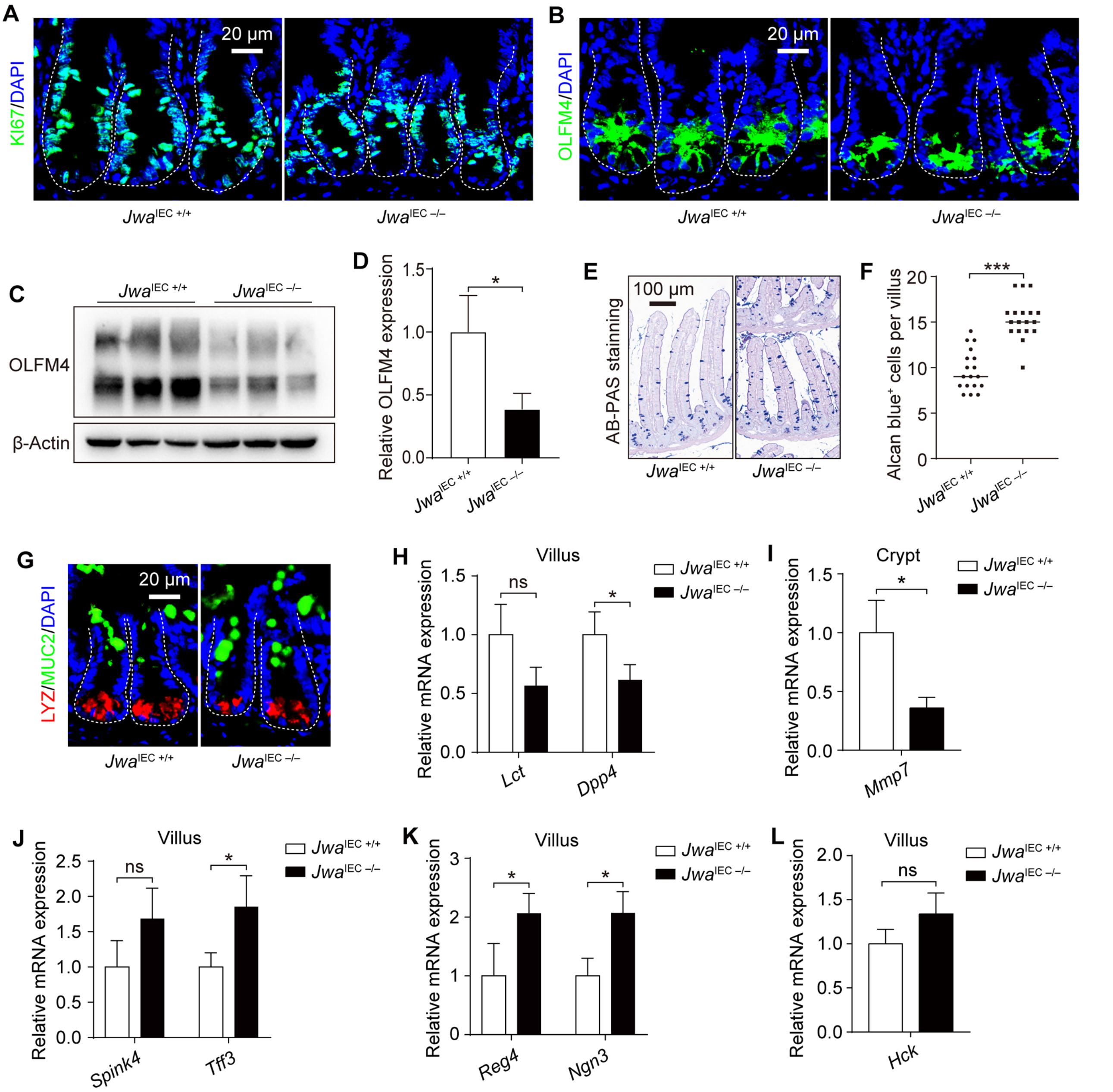

Supplementary Figure S4. Intestinal epithelial *Jwa* deletion reduces intestinal stem cells and skews the distributions of intestinal epithelial cells lineage. (A) Immunofluorescence staining of KI67 in the intestinal sections of 2-month-old $Jwa^{IEC\ +/+}$ and $Jwa^{IEC\ -/-}$ mice. **(B)** Immunofluorescence staining of OLFM4 in the intestinal sections of 2-month-old $Jwa^{IEC\ +/+}$ and $Jwa^{IEC\ -/-}$ mice. **(C, D)** Immunoblotting of OLFM4 **(C)** and relative OLFM4 levels in crypts **(D)** of 2-month-old $Jwa^{IEC\ +/+}$ and $Jwa^{IEC\ -/-}$ mice, n=3 for each genotype. **(E, F)** Alcian blue staining for the goblet cells **(E)** and AB positive cell counts in villus **(F)** of 2-month-old $Jwa^{IEC\ +/+}$ and $Jwa^{IEC\ -/-}$ mice, n=3 for each genotype.

(G) Immunofluorescence co-staining of LYZ and MUC2 in the intestinal sections of 2-month-old *Jwa*$^{IEC\ +/+}$ and *Jwa*$^{IEC\ -/-}$ mice. **(H-L)** QRT–PCR detection of absorption enterocytes marker *Lct* and *Dpp4* **(H)** in villi, Paneth cell marker *Mmp7* **(I)** in crypts, goblet cell marker *Spink4* and *Tff3* **(J)**, enteroendocrine cell marker *Reg4* and *Ngn3* **(K)**, and tuft cell marker *Hck* in villi **(L)** of 2-month-old *Jwa*$^{IEC\ +/+}$ and *Jwa*$^{IEC\ -/-}$ mice, n=3 for each genotype. ^{ns} No significance, *P < 0.05 and ***P < 0.001.

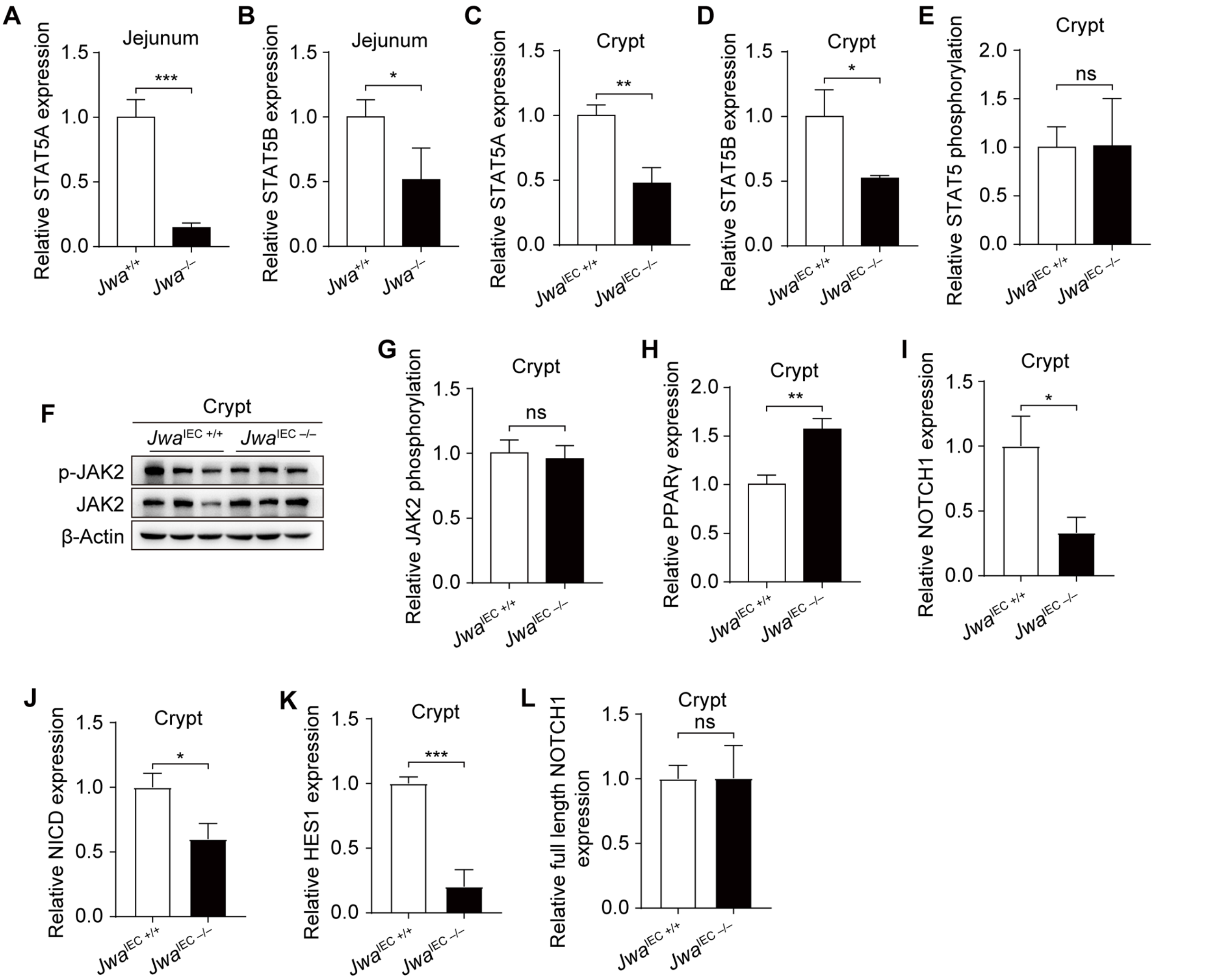

Supplementary Figure S5. Intestinal epithelial *Jwa* deletion disturbs PPARγ/STAT5 axis through inhibiting Notch signal pathway. (A, B) Relative STAT5A **(A)** and STAT5B **(B)** levels in the jejunum of 2-month-old Jwa$^{+/+}$ and Jwa$^{-/-}$ mice, n=3 for each genotype. **(C-E)** Relative STAT5A **(C)**, STAT5B **(D)** levels and relative STAT5 phosphorylation level **(E)** in crypts of 2-month-old *Jwa*$^{\mathrm{IEC}\ +/+}$ and *Jwa*$^{\mathrm{IEC}\ -/-}$ mice, n=3 for each genotype. **(F, G)** Immunoblotting of JAK2, p-JAK2 **(F)** and relative JAK2 phosphorylation level **(G)** in crypt of 2-month-old *Jwa*$^{\mathrm{IEC}\ +/+}$ and *Jwa*$^{\mathrm{IEC}\ -/-}$ mice, n=3 for each genotype. **(H-L)** Relative levels of PPARγ **(H)**, NOTCH1 **(I)**, NICD **(J)**, HES1 **(K)** and full length NOTCH1 **(L)** in crypts of 2-month-old *Jwa*$^{\mathrm{IEC}\ +/+}$ and *Jwa*$^{\mathrm{IEC}\ -/-}$ mice, n=3 for each genotype. ^{ns} No significance, *$P < 0.05$, **$P < 0.01$ and ***$P < 0.001$.

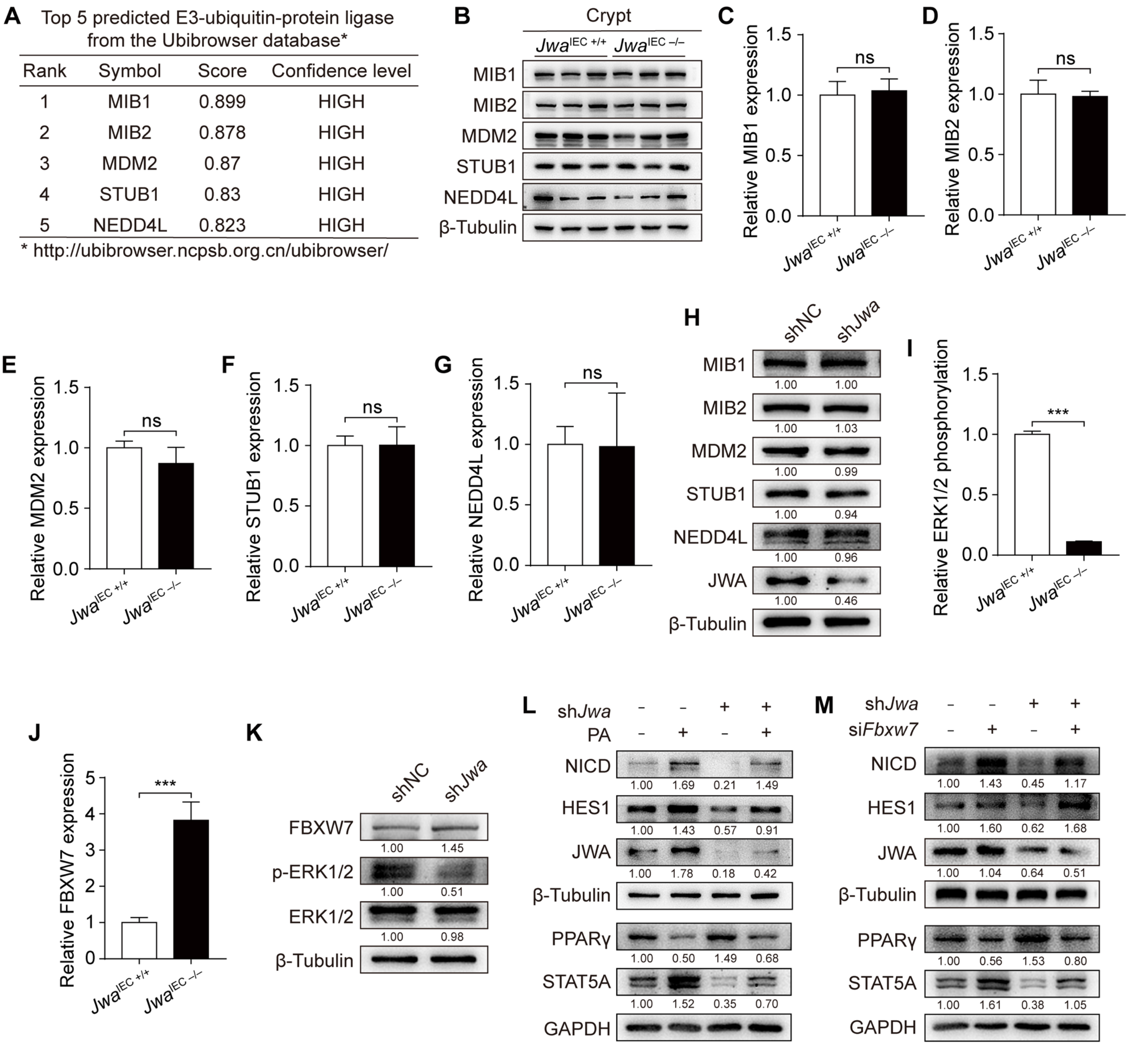

Supplementary Figure S6. *Jwa* deficiency promotes degradation of Notch1 through ERK/Fbxw7 axis. (A) The predicted top five E3 ubiquitin ligases target Notch1. **(B-G)** Immunoblotting of the predicted top five E3 ubiquitin ligases **(B)**, and relative MIB1 **(C)**, MIB2 **(D)**, MDM2 **(E)**, STUB1 **(F)** and NEDD4L **(G)** levels in crypts of 2-month-old *Jwa*$^{IEC +/+}$ and *Jwa*$^{IEC –/–}$ mice, n=3 for each genotype. **(H)** Immunoblotting of the predicted top five E3 ubiquitin ligases in IEC-6 cells transfected with sh*Jwa* plasmid, 3 independent replicates were carried out. **(I, J)** Relative ERK1/2 phosphorylation level **(I)** and FBXW7 level **(J)** in crypts of 2-month-old *Jwa*$^{IEC +/+}$ and *Jwa*$^{IEC –/–}$ mice, n=3 for each

genotype. **(K)** Immunoblotting of FBXW7, ERK1/2 and p-ERK1/2 in IEC-6 cells transfected with sh*Jwa* plasmid, 3 independent replicates were carried out. **(L)** Immunoblotting of JWA, NICD, HES1, PPARγ and STAT5A in IEC-6 cells transfected with sh*Jwa* plasmid followed by Pamoic acid (10 μM) treatment, 3 independent replicates were carried out. **(M)** Immunoblotting of JWA, NICD, HES1, PPARγ and STAT5A in IEC-6 cells co-transfected with sh*Jwa* and si*Fbxw7*, 3 independent replicates were carried out. ^{ns} No significance and $^{***}P < 0.001$.

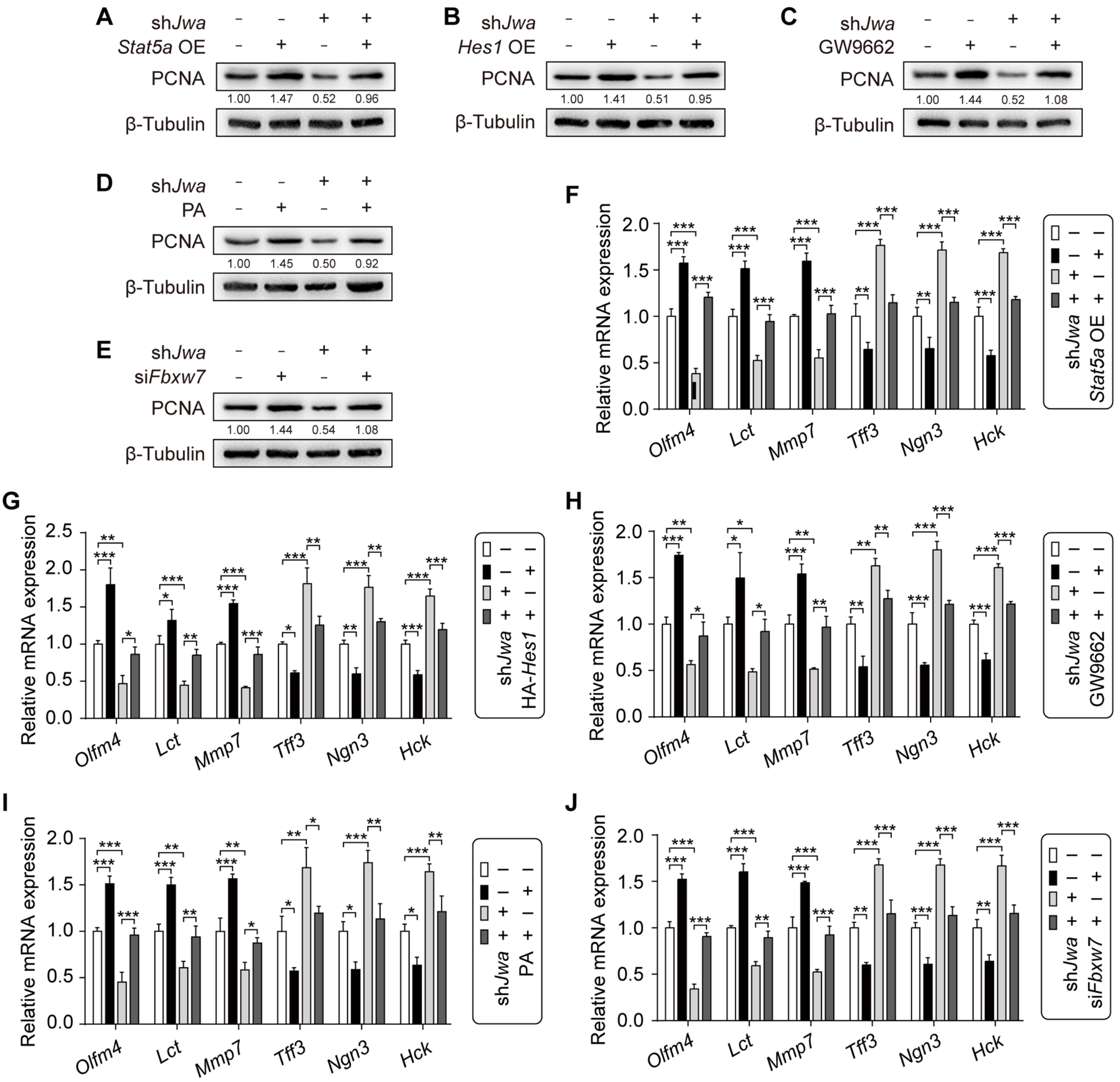

Supplementary Figure S7. Restoration of the ERK/FBXW7 and NOTCH1/PPARγ/STAT5 axes reverses *Jwa* deficiency-induced cellular phenotypic changes *in vitro*. (A-E) Immunoblotting of PCNA in IEC-cells co-tranfected with sh*Jwa* and *Stat5a* OE plasmids **(A)**, co-tranfected with sh*Jwa* and *Hes1* OE plasmids **(B)**, transfected with sh*Jwa* plasmid followed by GW9662 (10 μM) treatment **(C)**, transfected with sh*Jwa* plasmid followed by Pamoic acid (10 μM) treatment **(D)** or co-tranfected with sh*Jwa* and si*Fbxw7* **(E)**, 3 independent replicates were carried out. **(F-J)** QRT–PCR detection of ISC marker *Olfm4*, absorptive enterocyte marker *Lct*, Paneth cell marker *Mmp7*, goblet cell marker

Tff3, enteroendocrine cell marker *Ngn3* and tuft cell marker *Hck* in IEC-6 cell co-tranfected with sh*Jwa* and *Stat5a* OE plasmids **(F)**, co-tranfected with sh*Jwa* and *Hes1* OE plasmids **(G)**, transfected with sh*Jwa* plasmid followed by GW9662 (10 μM) treatment **(H)**, transfected with sh*Jwa* plasmid followed by Pamoic acid (10 μM) treatment **(I)** or co-tranfected with sh*Jwa* and si*Fbxw7* **(J)**, 3 independent replicates were carried out. **P* < 0.05, ***P* < 0.01 and ****P* < 0.001.

Supplementary Tables

Supplementary Table S1. Antibodies used in this study

Antibodies	Dilution	Manufacture	Catalog No.
anti-JWA mouse mAb	IB 1:100	Our laboratory	-
anti-JWA rabbit pAb	IF 1:50	Sigma Aldrich (St. Louis, MO, USA)	SAB1306837
anti-KI67 rabbit pAb	IHC 1:500	Servicebio (Wuhan, China)	GB111499
anti-BrdU mAb	IF 1:50	Proteintech (Wuhan, China)	66241-1-Ig
anti-OLFM4 rabbit mAb	IB 1:1000<br>IHC/IF 1:200	Cell Signaling Technology (Boston, MA, USA)	#39141
anti-LYZ rabbit mAb	IF 1:200<br>IHC 1:1000	Abcam (Cambridge, UK)	ab108508
anti-MUC2 rabbit pAb	IF 1:500	Servicebio (Wuhan, China)	GB11344
anti-Cleaved CASPASE-3 rabbit pAb	IF 1:500	Servicebio (Wuhan, China)	GB11532
anti-STAT5A rabbit pAb	IB 1:2000	Affinity (Changzhou, China)	AF6303
anti-STAT5B rabbit pAb	IB 1:2000	Affinity (Changzhou, China)	AF6340

Antibodies	Dilution	Manufacture	Catalog No.
anti-pY-STAT5 rabbit pAb	IB 1:1000	Bioworlde (Nanjing, China)	BS4185
anti-NOTCH1 rabbit pAb	IB 1:1000	Proteintech (Wuhan, China)	20687-1-AP
anti-NOTCH1 rabbit mAb	IB 1:2000 IP 1:200	Abclonal (Wuhan, China)	A19090
anti-Cleaved NOTCH1 (Val1744) [NICD] rabbit pAb	IB 1:1000	Cell Signaling Technology (Boston, MA, USA)	#4147
anti-HES1 rabbit pAb	IB 1:2000	Affinity (Changzhou, China)	AF7575
anti-PPARγ rabbit pAb	IB 1:5000	Proteintech (Wuhan, China)	16643-1-AP
anti-JAK2 rabbit pAb	IB 1:500	Wanleibio (Shenyang, China)	WL02188
anti-p-JAK2 rabbit pAb	IB 1:500	Wanleibio (Shenyang, China)	WL02997
anti-ERK1/2 mouse mAb	IB 1:2000	Affinity (Changzhou, China)	BF8004
anti-p-ERK1/2 rabbit pAb	IB 1:2000	Affinity (Changzhou, China)	AF1015

Antibodies	Dilution	Manufacture	Catalog No.
anti-FBXW7 rabbit pAb	IB 1:5000	Beyotime (Shanghai, China)	AF6873
anti-UBIQUITIN mouse mAb	IB 1:200	Santa cruz (Dallas, TX, USA)	sc-271289
anti-β-Tubulin mouse mAb	IB 1:5000	Abclonal (Wuhan, China)	AC021
anti-β-Actin mouse mAb	IB 1:1000	Servicebio (Wuhan, China)	GB12001
anti-GAPDH mouse mAb	IB 1:1000	Servicebio (Wuhan, China)	GB12002
HRP-labeled Goat Anti-Mouse IgG(H+L)	IB 1:1000<br>IHC 1:200	Beyotime (Shanghai, China)	A0216
HRP-labeled Goat Anti-Rabbit IgG(H+L)	IB 1:1000<br>IHC 1:200	Beyotime (Shanghai, China)	A0208
Alexa Fluor 488-conjugated Goat Anti-Mouse IgG H&L	IF 1:500	Abcam (Cambridge, UK)	ab150113
Alexa Fluor 488-conjugated Goat Anti-Rabbit IgG H&L	IF 1:500	Abcam (Cambridge, UK)	ab150077

Antibodies	Dilution	Manufacture	Catalog No.
Alexa Fluor 555-conjugated Goat Anti-Rabbit IgG H&L	IF 1:500	Abcam (Cambridge, UK)	ab150078

Supplementary Table S2. Target sequence of shRNAs and siRNA used in this study

Name	Specie	Target sequence
sh*Jwa* #1	Rat	5'-GACTTCGAAACCTCAAGAACA-3'
sh*Jwa* #2	Rat	5'-CCAGCTACTTCCTCATATCC-3'
sh*Jwa* #3	Rat	5'-TTGTTGATGTTCATCCATGC-3'
si*Fbxw7*	Rat	5'-ACAGGACAGTGTTTACAGA-3'

Supplementary Table S3. Primers used in QRT-PCR

Primer Name	Specie	Sequence
Jwa	Mouse	Forward: 5’-GGCAGCGCACAATAAAGACAT-3’<br>Reverse: 5’-ACACCCCCGAACATGGATATG-3’
Lgr5	Mouse	Forward: 5’-TAAAGACGACGGCAACAGTG-3’<br>Reverse: 5’-GCCTTCAGGTCTTCCTCAAA-3’
Lgr5	Rat	Forward: 5’-CGTAGGCAACCCTTCTCTTATC-3’<br>Reverse: 5’-GTAATTTGCGAGGCACCATTC-3’
Olfm4	Mouse	Forward: 5’-TGAAGGAGATGCAAAAACTGG-3’<br>Reverse: 5’-CTCCAGCTTCTCTACCAAGAGG-3’
Olfm4	Rat	Forward: 5’-TGAGCTCTACACATTCCCTGG-3’<br>Reverse: 5’-GTACCACCTTCTGTCCACGAC-3’
Smoc2	Mouse	Forward: 5’-GGAGCAGGGAAAGCAGATGAT-3’<br>Reverse: 5’-AACTTGCTCGGTCCAGAGTG-3’
Ascl2	Mouse	Forward: 5’-CCTCTCTCGGACCCTCTCTCAG-3’<br>Reverse: 5’-CAGTCAAGGTGTGCTTCCATGC-3’
Msi1	Mouse	Forward: 5’-CTCTCACCCCTGGACGATGG -3’<br>Reverse: 5’-TGGTACCCATTGGTGAAGGC -3’
Sis	Mouse	Forward: 5’-CGTTTCCGGTTCAAGCTCACA-3’<br>Reverse: 5’-CCTGATGACTTTGATGCTGAACG-3’
Sis	Rat	Forward: 5’-GACCCTGCCATCTCCATAAAT-3’<br>Reverse: 5’-CTGGCCATACCTCTCCAATAAG-3’

Primer Name	Specie	Sequence
Lct	Mouse	Forward: 5’-CTGTCATGGGCACAACTTCTC-3’<br>Reverse: 5’-TGTGGCATAATCAGCAAAGAGG-3’
Lct	Rat	Forward: 5’-CCAGGGAATGGTGTGAAAGA-3’<br>Reverse: 5’-TCAGGGTTCGAAGGATGTTTAG-3’
Dpp4	Mouse	Forward: 5’-CACCTCTGATGGAAGCAGCTTC-3’<br>Reverse: 5’-GATAATCGCTGGTCAGAGCTTCG-3’
Lyz1	Mouse	Forward: 5’-GGAATGGATGGCTACCGTGG-3’<br>Reverse: 5’-CATGCCACCCATGCTCGAAT-3’
Lyz	Rat	Forward: 5’-AGCTCAGCATGAGAGCAATTA-3’<br>Reverse: 5’-TGCCGTCATTACACCAGTATC-3’
Mmp7	Mouse	Forward: 5’-AGGAAGCTGGAGATGTGAGC-3’<br>Reverse: 5’-TCTGCATTTCCTTGAGGTTG-3’
Mmp7	Rat	Forward: 5’-CAGTGGACAAACTGAGGGAAA-3’<br>Reverse: 5’-CACCTGGGCTTCTGCATTAT-3’
Muc2	Mouse	Forward: 5’-GAAGCCAGATCCCGAAACCA-3’<br>Reverse: 5’-GAATCGGTAGACATCGCCGT-3’
Muc2	Rat	Forward: 5’-CGTCATCCCCTGGAACAACA-3’<br>Reverse: 5’-TTGGCCCTGTTGTGGTCTTT-3’
Spink4	Mouse	Forward: 5’-TGCAGTCACATAGCTCACAAG-3’<br>Reverse: 5’-CCATGCCAAGGAGGGGAA-3’

Primer Name	Specie	Sequence
Tff3	Mouse	Forward: 5’-TTGCTGGGTCCTCTGGGATAG-3’<br>Reverse: 5’-TACACTGCTCCGATGTGACAG-3’
Tff3	Rat	Forward: 5’-CCAGGAATTTGTTGGCCTATCT-3’<br>Reverse: 5’-GGTTGTTACACTGCTCTGATGT-3’
Gcg	Mouse	Forward: 5’-CTTCCCAGAAGAAGTCGCCA-3’<br>Reverse: 5’-GTGACTGGCACGAGATGTTG-3’
Gcg	Rat	Forward: 5’-AGAACCACTTGAAGACCCTAATC-3’<br>Reverse: 5’-GGAGTCCAGGTATTTGCTGTAG-3’
Ngn3	Mouse	Forward: 5’-TCTCAAGCATCTCGCCTCTTC-3’<br>Reverse: 5’-ACAGCAAGGGTACCGATGAGA-3’
Ngn3	Rat	Forward: 5’-CGGATGACGCCAAACTTACA-3’<br>Reverse: 5’-TAGAAGCTGTGGTCCGCTAT-3’
Reg4	Mouse	Forward: 5’-CTGAGCTGGAGTGTCAGTCAT-3’<br>Reverse: 5’-GTCCACTGCCATAATTGCTTCT-3’
Dclk1	Mouse	Forward: 5’-GGGTGAGAACCATCTACACCATC-3’<br>Reverse: 5’-CCAGCTTCTTAAAGGGCTCGAT-3’
Dclk1	Rat	Forward: 5’-CTCCTCCACTTCACTTTCATCC-3’<br>Reverse: 5’-TCTCCCGACTTTGTATCTCTCT-3’
Hck	Mouse	Forward: 5’-TCCTCCGAGATGGAAGCG-3’<br>Reverse: 5’-ACAGTGCGACCACAATGGTAT-3’

Primer Name	Specie	Sequence
Hck	Rat	Forward: 5’-CGTATGCCTCGACCAGATAAC-3’<br>Reverse: 5’-AGCACGCTCTGGATGTATTC-3’
Cpt1a	Mouse	Forward: 5’-GGCATAAACGCAGAGCATTCCTG-3’<br>Reverse: 5’-CAGTGTCCATCCTCTGAGTAGC-3’
Cpt2	Mouse	Forward: 5’-GATGGCTGAGTGCTCCAAATACC-3’<br>Reverse: 5’-GCTGCCAGATACCGTAGAGCAA-3’
Cd36	Mouse	Forward: 5’-GGACATTGAGATTCTTTTCCTCTG-3’<br>Reverse: 5’-GCAAAGGCATTGGCTGGAAGAAC-3’
Stat5a	Mouse	Forward: 5’-CCGAAACCTCTGGAATCTGA-3’<br>Reverse: 5’-ACGAACTCAGGGACCACTTG-3’
Stat5b	Mouse	Forward: 5’-GTGAAGCCACAGATCAAGCA-3’<br>Reverse: 5’-TCGGTATCAAGGACGGAGTC-3’
PPARγ	Mouse	Forward: 5’-ATTGAGTGCCGAGTCTGTGG-3’<br>Reverse: 5’-GGCATTGTGAGACATCCCCA-3’
Notch1	Mouse	Forward: 5’-GCTGCCTCTTTGATGGCTTCGA-3’<br>Reverse: 5’-CACATTCGGCACTGTTACAGCC-3’
Hes1	Mouse	Forward: 5’-CCAGCCAGTGTCAACACGA-3’<br>Reverse: 5’-AATGCCGGGAGCTATCTTTCT-3’
Hes2	Mouse	Forward: 5’-CTGAAGGGTCTCGTATTGCCG-3’<br>Reverse: 5’-CGCAGGTGCTCTAGTAGGC-3’

Primer Name	Specie	Sequence
Hes5	Mouse	Forward: 5’-AGTCCCAAGGAGAAAAACCGA-3’<br>Reverse: 5’-GCTGTGTTTCAGGTAGCTGAC-3’
Hes6	Mouse	Forward: 5’-ACCACCTGCTAGAATCCATGC-3’<br>Reverse: 5’-GCACCCGGTTTAGTTCAGC-3’
Hes7	Mouse	Forward: 5’-CGGGAGCGAGCTGAGAATAG-3’<br>Reverse: 5’-CACGGCGAACTCCAGTATCT-3’
Actb	Mouse	Forward: 5’-AGATGTGGATCAGCAAGCA-3’<br>Reverse: 5’-GCGCAAGTTAGGTTTTGTCA-3’
Actb	Rat	Forward: 5’-CCGCGAGTACAACCTTCTTGC-3’<br>Reverse: 5’-TCGTCATCCATGGCGAACTGG-3’

Supplementary Table S4. Down-regulated proteins in jejunum tissues induced by *Jwa* deletion (Fold Change: *Jwa*$^{-/-}$ vs *Jwa*$^{+/+}$)

Protein Name	Fold Chage	Regulation	Rank
FLNA	0.0013	Down	1
STAT5A	0.0013	Down	2
RHBDL2	0.0018	Down	3
PLEKHH2	0.0018	Down	4
MARCKS	0.0020	Down	5
MRE11A	0.0020	Down	6
PLA2G12B	0.0022	Down	7
MGST1	0.0022	Down	8
ENO2	0.0024	Down	9
SRP19	0.0027	Down	10
TBC1D2	0.0031	Down	11
HAUS3	0.0032	Down	12
MRPS34	0.0033	Down	13
NAA10	0.0036	Down	14
JTB	0.0042	Down	15
NUPR1	0.0042	Down	16
PKIG	0.0049	Down	17
GNG12	0.0189	Down	18
METTL7B	0.0287	Down	19

Protein Name	Fold Chage	Regulation	Rank
MYADM	0.0288	Down	20
TRMT61A	0.0329	Down	21
ELF1	0.0373	Down	22
GNAO1	0.0470	Down	23
STAT5B	0.0596	Down	24
CHIA1	0.0619	Down	25
NT5C	0.0648	Down	26
KLHL9	0.0663	Down	27
BMI1	0.0690	Down	28
CYP1A2	0.0698	Down	29
CD93	0.0728	Down	30
ANKIB1	0.0871	Down	31
NRP1	0.0968	Down	32
UTP18	0.1901	Down	33
ARL6IP5	0.3085	Down	34
KRT2	0.5104	Down	35
LCTL	0.6273	Down	36
AKR1B7	0.6641	Down	37

Supplementary Table S5. Predicted transcript factors bind to the *Pparg* promotor

Matrix ID	Name	Score	Relative score	Start	End	Strand	Predicted sequence
MA1099.2	HES1	14.4264	0.999430951	704	713	+	GGCACGTGCC
MA1099.2	HES1	14.4264	0.999430951	704	713	-	GGCACGTGCC
MA0616.2	HES2	16.1882	1.000000009	704	713	+	GGCACGTGCC
MA0616.2	HES2	16.1882	1.000000009	704	713	-	GGCACGTGCC
MA0821.1	HES5	18.7952	0.978104532	703	714	-	TGGCACGTGCCT
MA0821.1	HES5	18.719	0.977138462	703	714	+	AGGCACGTGCCA
MA1493.1	HES6	12.8217	0.920264948	704	713	+	GGCACGTGCC
MA1493.1	HES6	12.8217	0.920264948	704	713	-	GGCACGTGCC
MA0822.1	HES7	19.0547	0.97173189	703	714	+	AGGCACGTGCCA
MA0822.1	HES7	18.6492	0.966723384	703	714	-	TGGCACGTGCCT
